# Decentralized Tele-Rehabilitation via Edge AI-Oracle Architecture for Spatiotemporal Pain Assessment

**DOI:** 10.3390/s26134136

**Published:** 2026-07-01

**Authors:** Nataliya Bilous, Danylo Ostapchenko, Iryna Ahekian, Marcus Frohme

**Affiliations:** 1 Computer Science Faculty, Kharkiv National University of Radio Electronics, 14 Nauky ave., 61166 Kharkiv, Ukraine; danylo.ostapchenko@nure.ua (D.O.); iryna.ahekian@nure.ua (I.A.); 2 Division Molecular Biotechnology and Functional Genomics, Technical University of Applied Sciences, 1 Hochschulring, 15745 Wildau, Germany; mfrohme@th-wildau.de

**Keywords:** tele-rehabilitation, Edge AI, smart contracts, pain assessment, computer vision, IPFS, Oracle problem, Trusted Execution Environment, LSTM, MediaPipe

## Abstract

Remote tele-rehabilitation requires objective pain assessment, but existing approaches fail in two distinct ways. Self-report scales such as the Visual Analog Scale and the Numeric Pain Rating Scale are easy to falsify, opening a special case of the Oracle problem in blockchain-based insurance. Cloud-based computer vision handles falsification but transmits raw biometric video off the patient’s device, violating privacy requirements. A decentralized Edge AI-Oracle architecture is proposed that combines MediaPipe Face Mesh landmark extraction with a recurrent classifier mapping Action-Unit feature sequences to a learned pain score aligned with the Prkachin and Solomon Pain Intensity scale. The recurrent cell is selected empirically across short-context (T = 2) and long-context (T = 120 frames at 24 fps) regimes, with a two-layer Long Short-Term Memory (LSTM) network adopted for deployment. Inference and Elliptic Curve Digital Signature Algorithm (ECDSA) signing run inside an ARM TrustZone Trusted Execution Environment (TEE). Biometric logs are stored off-chain on the InterPlanetary File System (IPFS). Smart contracts anchor results on-chain and open a 24 h optimistic verification window for an off-chain Watchtower auditor. On SynPAIN the LSTM reaches F1 = 0.683 on T = 120 video (leave-one-stratum-out), with a directional but non-significant advantage over Gated Recurrent Unit (GRU) (Wilcoxon *p* = 0.167). Cross-dataset validation on BioVid Heat Pain Database Part A (87 subjects, 174 paired observations, leave-one-subject-out) yields F1 = 0.519 for LSTM and 0.499 for GRU (Wilcoxon *p* = 0.549). A processor-only TEE surrogate benchmark estimates 1.96 ms (FP32) and 0.45 ms (INT8) inference latency at T = 120 with a 0.34 MB footprint and 707 µs ECDSA signing latency, leaving the INT8 inference latency more than an order of magnitude below the 33 ms per-frame budget. The dual-layer storage reduces gas costs by a factor of 23.4 (160,261 vs. 3,744,872 gas), corresponding to an illustrative mainnet cost of approximately 0.53 USD per submission at 1 gwei, rising to roughly 16 USD at a busier 30 gwei, and falling to approximately 0.005 USD on Arbitrum One (April 2026 reference parameters), so that continuous monitoring is economically practical on Layer-2. An adaptive-adversary analysis of the Watchtower shows that gross score tampering is detected at every usable operating threshold, whereas a rational adversary who inflates by less than the dispute threshold, or who shapes the injected score to fall just inside it, evades detection. Because the false-positive rate reaches zero only for δ≳0.15, the protocol bounds rather than eliminates patient-side fraud and motivates a zero-knowledge proof-of-inference successor. The framework is architecturally and economically feasible as a cryptographically verifiable, privacy-preserving tele-rehabilitation substrate aligned with General Data Protection Regulation (GDPR) and Health Insurance Portability and Accountability Act (HIPAA) requirements through the Zero-Video Transmission principle, while remaining economically viable under post-Dencun mainnet and Layer-2 conditions. Recognition accuracy on real-world data and robustness to small-magnitude tampering remain limitations that the interchangeable recognition and audit components must improve before clinical deployment.

## 1. Introduction

Modern healthcare and rehabilitative medicine are undergoing a stage of rapid digital transformation within the Health 4.0 paradigm, with demand for telemedicine and remote physical rehabilitation services rising sharply during the COVID-19 pandemic and remaining well above pre-pandemic levels since [1], driven by the need to reduce clinical costs and improve patient access to specialized care. Central to this process is the transition from periodic in-person clinical visits to continuous remote monitoring of the patient’s state. However, the effectiveness of remote therapy remains critically dependent on the accuracy and objectivity of clinical data, particularly regarding the intensity of the pain syndrome experienced by the patient during exercise sessions.

Traditionally, pain assessment in clinical practice relies on subjective self-report tools, such as the Visual Analog Scale (VAS) and the Numeric Pain Rating Scale (NPRS) [2,3]. While these methods are easy to implement, they are prone to significant cognitive biases and intentional falsification when applied in unsupervised remote settings. In the context of decentralized health insurance and automated payout systems based on smart contracts, this creates a fundamental Oracle problem: the inability of a blockchain-based system to verify the authenticity of biological data originating outside the network [4,5]. A patient may intentionally overstate the perceived pain level to obtain higher insurance compensation or unauthorized access to controlled analgesics, undermining the economic stability of decentralized medical protocols.

To address the subjectivity issue, recent research has focused on computer vision (CV) methods for objective pain estimation through facial expression analysis. State-of-the-art models utilize convolutional architectures to detect Action Units (AUs) associated with the Prkachin and Solomon Pain Intensity (PSPI) scale [6,7]. Despite their high recognition accuracy, these systems rely on cloud-centric architectures where raw video streams are transmitted to centralized servers for inference. This approach creates a significant Privacy Gap because biometric video data is protected under strict regulations such as the General Data Protection Regulation (GDPR) and the Health Insurance Portability and Accountability Act (HIPAA) [8]. The risk of biometric data leakage makes centralized CV solutions problematic for widespread medical adoption.

Facial landmarks provide a compact and privacy-preserving sensing representation for affective and physiological state estimation. Unlike raw facial video streams, landmark-based representations significantly reduce sensitive visual information while preserving essential spatiotemporal patterns associated with pain-related facial activity. This makes landmark sensing particularly suitable for real-time edge deployment in tele-rehabilitation systems operating under bandwidth, latency, and privacy constraints.

Migrating the inference workload to the edge of the network introduces a new class of security vulnerabilities. In a standard mobile operating system (OS) environment, a malicious user with administrative privileges can intercept the application memory, decompile the inference engine, or substitute the trained weights of the neural network to generate fraudulent pain indices [9,10]. The development of a solution that is simultaneously private (aligned with GDPR requirements at the data-flow level), objective (independent of patient self-report) and resilient to client-side tampering (suitable for integration with smart contracts) therefore remains an open scientific challenge.

The object of study is the process of cryptographically verifiable pain assessment in decentralized tele-rehabilitation systems. The subject of study comprises the methods of spatiotemporal facial expression analysis, hardware-isolated inference, and decentralized audit applied to this process. The purpose of this work is to increase the reliability and confidentiality of remote pain assessment by developing a decentralized Edge Artificial Intelligence (AI)-Oracle architecture that combines on-device MediaPipe-Long Short-Term Memory (LSTM) inference, Trusted Execution Environment (TEE)-based cryptographic attestation, and an asynchronous off-chain audit mechanism.

To achieve the stated goal, the following tasks are addressed:

- formalize the problem of objective and tamper-resistant pain assessment in untrusted client environments;
- design a spatiotemporal pipeline that combines MediaPipe Face Mesh landmark extraction with a recurrent temporal classifier, empirically selecting the recurrent architecture among LSTM and Gated Recurrent Unit (GRU) candidates and benchmarking against feed-forward baselines, capable of real-time PSPI estimation on a mobile edge device;
- develop a hardware-isolated execution model that decomposes the inference workload between the Rich OS and the Trusted Execution Environment, providing cryptographic attestation of the result;
- design a decentralized data flow consisting of off-chain storage of biometric logs in the InterPlanetary File System (IPFS), on-chain anchoring through an Ethereum Virtual Machine (EVM)-compatible smart contract, and an asynchronous Watchtower auditor with a Timelock-based dispute window.

The remainder of this paper is organized as follows. The rest of this introduction reviews related work on tele-rehabilitation platforms, automated pain recognition, hardware-assisted security, and blockchain-anchored medical pipelines, and positions the present contribution against this background. Section 2 formalizes the four architectural constraints (clinical objectivity, privacy, authenticity, and economic feasibility), then describes the proposed architecture, the spatiotemporal pipeline, the TEE decomposition, the cryptographic attestation protocol, the IPFS-anchored evidence layer, and the smart contract state machine, together with the experimental protocol on the SynPAIN and BioVid corpora. Section 3 reports the recognition, on-device, on-chain, and adversarial-tampering results. Section 4 places the results in the context of prior work and discusses the limitations. Section 5 concludes.

The digital transformation of rehabilitative medicine has produced a generation of musculoskeletal (MSK) platforms that aim to deliver clinical-grade therapy outside traditional hospital settings. Leading commercial solutions such as Kaia Health, SWORD Health, and Hinge Health have established a benchmark for remote physical therapy by demonstrating that computer vision and sensor-based tracking can achieve outcomes comparable to human-led interventions [11,12,13]. Kaia Health utilizes on-device computer vision for exercise form correction but does not verify the patient’s physiological response such as pain intensity. SWORD Health employs wearable Inertial Measurement Units (IMUs) to capture joint angles, but the reliance on external hardware increases the attack surface and creates logistical barriers for patients with limited mobility. Hinge Health combines computer vision with deep clinical integration into corporate insurance programs but follows the same centralized data ownership model. None of these platforms provides a transparent decentralized audit trail of the session data, which makes them vulnerable to administrative fraud at the database level by personnel of the service provider or by external attackers who have compromised the cloud back end. Beyond commercial platforms, recent academic work has explored dual-stream architectures that jointly model facial reactions and skeletal motion during physical exercise from monocular RGB video [14], reinforcing the methodological case for sensor-free rehabilitation monitoring based exclusively on a single camera and complementing the present work, which concentrates on the facial stream under cryptographic attestation.

The recent academic literature has shifted focus toward objective pain assessment as a replacement for subjective scales such as VAS and NPRS [2]. The PSPI metric remains the de facto gold standard for automated detection [7]. Methodological advancements in tracking facial reference points have significantly improved the precision of landmark-based state determination [15,16]. The present work extends this line of inquiry from static head-pose tracking into the temporal-classification regime, where landmark sequences are processed by a recurrent classifier to capture the dynamic onset–apex–offset structure of pain micro-expressions. The emphasis on modeling temporal dynamics from consecutive frames, rather than single-frame static features, is shared more broadly across video understanding, including work on universal moving object segmentation that learns temporal pixel distributions under a lightweight, deployment-oriented design [17]. Spatiotemporal models such as LSTM networks demonstrate superior performance on facial pain expression datasets, because they capture the dynamic onset–apex–offset structure of pain micro-expressions, which static convolutional networks fail to discriminate from incidental facial muscle contractions [7]. Earlier methodological surveys of facial expression analysis for clinical health assessment [16] have identified frame-sequence classification as a viable approach for downstream pain detection, while their own evaluation focused on static-image classifiers. Recent studies have confirmed this trend: a customized spatial-temporal attention LSTM trained on a clinical pain corpus from 200 surgical patients [18] reported strong identification of significant pain levels using MediaPipe-based landmark extraction, and multi-modal frameworks combining MediaPipe with Bi-LSTM architectures have been deployed on edge hardware for real-time pain estimation [19]. Both approaches, however, assume a client-side environment and do not address tamper resistance or decentralized audit. Lightweight on-device perception frameworks, in particular MediaPipe Face Mesh [20], have made it computationally feasible to extract 478 three-dimensional facial landmarks at 30 frames per second (fps) on commodity smartphones, opening the possibility of moving the entire inference pipeline to the edge.

Despite these advancements, a critical Trust Gap persists. Existing CV-based pain assessment studies typically assume a benign client-side environment in which the video input and the model weights are authentic [6,16,18]. In a decentralized ecosystem where medical records trigger automated insurance payouts through smart contracts, this assumption is invalid, because an immutable blockchain cannot guarantee the truthfulness of external data that has already been tampered with before reaching the ledger [4,5]. Centralized AI solutions in turn violate the Zero-Video Transmission principle required for GDPR and HIPAA compliance [8]. Existing blockchain-based health record systems concentrate primarily on data sharing and interoperability across institutions [21,22] rather than on the secure generation of clinical data at the edge, and recent hybrid TEE/zero-knowledge architectures for medical AI [23] address verifiable federated training rather than real-time single-device inference. There is therefore a clear need for a framework that provides proof of inference, that is, a cryptographic guarantee that a specific untampered model has been executed on a legitimate biometric stream. TEEs such as ARM TrustZone [24,25] and Elliptic Curve Digital Signature Algorithm (ECDSA)-based attestation primitives [26] supply mature, mobile-keystore-available building blocks for this purpose that natively interoperate with EVM blockchains through the secp256k1 curve. The application of these primitives to low-latency facial expression analysis for tele-rehabilitation, combined with an off-chain audit protocol that detects model tampering after the fact, remains underexplored.

Within the Edge AI literature, MobileNet family architectures [27] have established themselves as the de facto efficiency benchmark for on-device computer vision, achieving favourable accuracy–latency trade-offs through depthwise-separable convolutions. Among recurrent alternatives for temporal sequence modeling, GRU networks [28] match LSTM performance on most short-sequence tasks while reducing the parameter count by roughly a quarter. Both architectural families therefore constitute relevant comparison points for the proposed pipeline: MobileNet as a true edge-class baseline (in contrast to the cloud-class ResNet-50 considered in earlier work), and GRU as a temporal-modeling alternative whose competitive standing relative to LSTM justifies an empirical rather than an a priori choice of recurrent cell.

A comparative analysis against the existing methodological landscape reveals consistent gaps. The following qualitative assessment rates each method family along three axes (privacy, objectivity, tamper resistance) according to whether raw biometric data leaves the device (privacy), whether the pain estimate derives from physiological markers rather than self-report (objectivity), and whether the pipeline resists client-side modification (tamper resistance). Under these criteria, traditional electronic patient-reported outcome systems rate high on privacy but provide no objectivity and no tamper resistance, because they record self-reported scores locally. Cloud-based CV platforms rate high on objectivity but low on privacy, because they transmit raw video off the device. Wearable IMU systems rate high on objectivity but only medium on tamper resistance, because the external sensor hardware enlarges the attack surface. None of the three categories provides a transparent decentralized audit trail or native compatibility with smart-contract-based insurance protocols. The proposed Edge AI-Oracle architecture simultaneously achieves high objectivity through MediaPipe-LSTM inference, high privacy through Zero-Video Transmission, high tamper resistance through TEE-attested cryptographic signatures, decentralized audit transparency through IPFS and on-chain anchoring, and native compatibility with smart-contract insurance, while reusing the built-in TEE of contemporary mobile devices without additional hardware overhead.

## 2. Materials and Methods

The proposed Edge AI-Oracle architecture is a layered system that resolves the simultaneous requirements of objectivity, privacy, tamper resistance, and economic feasibility. This section first formalizes the four architectural constraints, then describes the on-device perception and classification pipeline, the hardware-isolated execution model, the ECDSA-based attestation protocol, the IPFS-anchored evidence layer with the smart-contract state machine and the asynchronous Watchtower auditor, and finally the evaluation corpora (SynPAIN and BioVid Heat Pain Database, Part A) together with the four-experiment validation protocol.

The primary challenge in decentralized tele-rehabilitation is the absence of a reliable mechanism to bridge physical reality and digital records without compromising patient confidentiality. Let $P_{actual}\in [0,10]$ denote the true physiological pain intensity of the patient, and let $P_{\mathrm{reported}}\in [0,10]$ denote the value submitted to the medical or insurance system. In the traditional self-report paradigm, the reported value is contaminated by subjective and adversarial components:

(1) $$P_{\mathrm{reported}}=P_{\mathrm{actual}}+\varepsilon_{\mathrm{subj}}+\varepsilon_{\mathrm{bias}},$$

where $\varepsilon_{\mathrm{subj}}$ is the random error caused by the cognitive and emotional state of the patient, and $\varepsilon_{\mathrm{bias}}$ is the systematic bias, which in unsupervised remote settings may take the form of conscious symptom exaggeration motivated by financial incentives, a phenomenon documented in the clinical literature on secondary-gain and malingering in pain reporting [2,4,29]. The objective of any verifiable assessment system is to construct an estimator $\hat{P}$ such that the discrepancy $\left|\hat{P}-P_{\mathrm{actual}}\right|\to min$ while satisfying a set of additional constraints described below.

**Constraint 1 (Privacy).**  *The privacy requirement is imposed by GDPR and HIPAA. Let* $V_{local}$ *denote the raw video stream captured by the patient’s device, and let* Net *denote the public network. The privacy condition* $C_{priv}$ *is defined as:*

(2) $$C_{\mathrm{priv}}=1,\;\mathrm{if}\;V_{\mathrm{local}}\notin \mathrm{Net};\;C_{\mathrm{priv}}=0\;\mathrm{otherwise},$$

*Any architecturally acceptable solution must guarantee* $C_{\mathrm{priv}}=1$ *throughout the entire rehabilitation session.*

**Constraint 2 (Clinical objectivity).**  *The estimator* $\hat{P}$ *must be derived from observable physiological markers rather than from patient self-report. According to the Facial Action Coding System (FACS), pain intensity is encoded by a specific combination of facial AUs, and the PSPI metric is defined as:*

(3) $$PSPI=AU_{4}+\max\left(AU_{6},AU_{7}\right)+\max\left(AU_{9},AU_{10}\right)+AU_{43},$$

*where the values* AU4, AU6, AU7, AU9, AU10, AU43 *in the canonical PSPI definition denote FACS Action Unit intensities on the A–E scale [6]. In the proposed pipeline, these intensities are approximated by geometric proxies, namely Euclidean distances between anatomically meaningful landmark pairs, as defined in Equation (5). The use of inter-landmark distances as proxies for AU activation follows the established geometric-feature line of facial-expression analysis [15,16,30], in which the contraction and relaxation of facial muscles underlying each Action Unit manifest as measurable displacements between landmark positions. The estimator $\hat{P}$ is therefore a learned function of these geometric proxies rather than a direct PSPI computation, and the biomechanical validity of this approximation is verified empirically through the feature-level sanity check reported in Section 3, where the inter-class distance differences align with the directions predicted by the canonical PSPI literature.*

**Constraint 3 (Authenticity).**  *Let* $F_{\inf}:V_{\mathrm{local}}\to \hat{P}$ *denote the inference function executed on the client device, and let $\mathrm{Auth}\left(F_{\inf}\right)\in \{0,1\}$ denote a cryptographic attestation that $F_{\inf}$ was executed on legitimate hardware with unmodified model weights. For integration with smart contracts in a decentralized insurance scheme, the system must guarantee $\mathrm{Auth}\left(F_{\inf}\right)=1$ , otherwise the blockchain ledger will record an externally validated but internally falsified value (the classical “Garbage In, Garbage Out” failure mode of blockchain oracles [4,5]).*

**Constraint 4 (Economic feasibility).**  *Let* $G\left(\tau \right)$ *denote the total gas cost of recording one assessment transaction* τ *on an EVM-compatible blockchain. For continuous monitoring during a multi-week rehabilitation course,* $G\left(\tau \right)$ *must remain below a clinically acceptable threshold* $G_{max}$ *, which excludes naive on-chain storage of high-dimensional biometric logs.*

Formally, given the input video stream $V_{\mathrm{local}}$, the patient’s wallet identifier $id_{\mathrm{pat}}$, and the smart contract endpoint SC, the problem is to construct a tuple $\left\langle F_{inf},\sigma ,cid\right\rangle$ such that $\hat{P}=F_{\inf}\left(V_{\mathrm{local}}\right)$, $\sigma ={\mathrm{Sign}}_{\mathrm{TEE}}\left(\hat{P}\;|\;\mathrm{cid}\;|\;id_{\mathrm{pat}}\right)$, $\mathrm{cid}=\mathrm{IPFS}.\mathrm{put}\left(\log\left(V_{\mathrm{local}}\right)\right)$, and the smart contract submission $\mathrm{SC}.\mathrm{submit}\left(\hat{P},\;\mathrm{cid},\;\sigma \right)$ is accepted if and only if $\mathrm{Verify}\left(\sigma ,\;\hat{P},\;\mathrm{cid},\;id_{\mathrm{pat}}\right)=1$, subject to $C_{\mathrm{priv}}=1$, $\mathrm{Auth}\left(F_{\inf}\right)=1$, $G\left(\tau \right)\leq G_{\max}$, and $\left|\hat{P}-P_{\mathrm{actual}}\right|\to min$. The architecture described in the remainder of this section satisfies these four constraints simultaneously.

The Edge AI component performs the transformation $F_{\inf}:V_{\mathrm{local}}\to \hat{P}$. To satisfy the privacy constraint $C_{\mathrm{priv}}=1$, the raw video stream $V_{\mathrm{local}}$ is consumed in-place from the device camera and never persisted to non-volatile storage or transmitted over the network. The pipeline operates in three sequential stages: spatial landmark extraction, feature vector formation, and temporal classification.

In the first stage, the input video stream is processed at 30 fps by the MediaPipe Face Mesh framework [20], which produces a dense mesh of 478 three-dimensional landmarks $L_{t}=\{\left(x_{i},\;y_{i},\;z_{i}\right){\}}_{i=1,\ldots ,478}$ for each frame t. The raw pixel coordinates are mapped to a frame-normalized coordinate system relative to the centre of the video frame. Alternative open-source facial expression analysis toolkits, most prominently Py-Feat [31], integrate landmark detection with built-in Action Unit and emotion classifiers based on pretrained convolutional backbones. MediaPipe Face Mesh is preferred in the present pipeline because its inference graph is explicitly optimized for mobile deployment through the TensorFlow Lite delegate and produces a denser landmark set (478 vs. 68 in the canonical Py-Feat configuration), which is required for the geometric proxy computation defined in Equation (5).

(4) $$\hat{x_{l}}=\frac{x_{i}-w/2}{w},\;\hat{y_{l}}=\frac{y_{i}-h/2}{h},\;\hat{z_{l}}=\frac{z_{i}}{w},$$

where (w,h) are the width and height of the input frame in pixels. The normalization in Equation (4) eliminates the dependence of the feature vector on the absolute pixel coordinates and on the input resolution, which is critical because the position and orientation of the device relative to the patient’s face cannot be standardized in a home rehabilitation setting. Two of the forty-two landmark pairs, contributing to AU7 and AU43, rely on the iris reference points provided only by the 478-point FaceLandmarker topology, which is the reason this topology is adopted in preference to the legacy 468-point mesh.

In the second stage, a subset of anatomically relevant points corresponding to the AUs of the FACS taxonomy is selected from the 478 normalized landmarks. For each pair of landmarks $\left(x_{a},\;x_{b}\right)$ associated with an AU, the Euclidean distance is computed:

(5) $$d_{a,b}\left(t\right)=\sqrt{{\left(\hat{x_{a}}-\hat{x_{b}}\right)}^{2}+{\left(\hat{y_{a}}-\hat{y_{b}}\right)}^{2}+{\left(\hat{z_{a}}-\hat{z_{b}}\right)}^{2}},$$

For each of the six AUs relevant to the PSPI metric (AU4,AU6,AU7,AU9,AU10,AU43), seven Euclidean distances between anatomically meaningful landmark pairs are computed. The resulting feature vector $x_{t}\in R^{42}$ represents the spatial configuration of the face at frame t and serves as the input to the temporal classifier. The complete enumeration of these six Action Units, their seven constituent landmark pairs in the MediaPipe 478-point FaceLandmarker indexing, and the anatomical structure sampled by each inter-landmark distance is given in Table A1 (Appendix A).

In the third stage, a sliding window of length T frames is used to buffer the most recent feature vectors. The window length is a deployment parameter. As the temporal-sensitivity analysis in Section 3 shows, recognition accuracy is highest at T = 90 frames on the SynPAIN video corpus, but T = 120 frames is adopted for the production video regime because it yields a 5 s context at 24 fps that is directly comparable to the 4.8 s context used in the BioVid cross-dataset evaluation, over which the onset–apex–offset structure of a pain micro-expression unfolds:

(6) $$X_{t}=\left(x_{t-T+1},\;x_{t-T+2},\;\ldots ,\;x_{t}\right),$$

The buffered window is fed into a two-layer LSTM network [32], which models the onset, apex, and offset phases of a pain micro-expression. At each time step t, the LSTM cell updates its internal state according to the canonical gating equations:

(7) $$f_{t}=\sigma \left(W_{f}\cdot \left[h_{t-1},x_{t}\right]+b_{f}\right)\;i_{t}=\sigma \left(W_{i}\cdot \left[h_{t-1},x_{t}\right]+b_{i}\right)\;\tilde{C_{t}}=\tanh\left(W_{C}\cdot \left[h_{t-1},x_{t}\right]+b_{C}\right)\;C_{t}=f_{t}⊙C_{t-1}+i_{t}⊙\tilde{C_{t}}\;o_{t}=\sigma \left(W_{o}\cdot \left[h_{t-1},x_{t}\right]+b_{o}\right)\;h_{t}=o_{t}⊙\tanh\left(C_{t}\right)$$

where $f_{t},\;i_{t},\;o_{t}$ are the forget, input and output gates, respectively, $C_{t}$ is the cell state, $h_{t}$ is the hidden state, $\sigma \left(\cdot \right)$ denotes the logistic sigmoid activation, $\tanh\left(\cdot \right)$ is the hyperbolic tangent, ⊙ denotes the Hadamard product, and W, b are the trainable weight matrices and bias vectors. The hidden state of the second LSTM layer is passed to a fully connected dense layer followed by a sigmoid activation that produces a continuous pain score $\hat{P}\in \left[0,\;1\right]$, rounded to a binary outcome prior to on-chain submission to align with the integer storage type used by the smart contract.

The selection of a hybrid landmark-plus-recurrent architecture rather than a monolithic end-to-end convolutional network is dictated by the resource budget of the target edge device. End-to-end ResNet-50-class architectures consume in excess of 1 GB of memory and reduce the throughput to 10–15 fps on commodity smartphones [7], which is insufficient for capturing micro-expressions whose duration may be as short as 1/25 of a second. The proposed hybrid pipeline delegates the computationally expensive perception task to the highly optimized MediaPipe inference graph and reserves the recurrent classifier only for the lightweight 42-dimensional feature stream. The choice of recurrent cell (LSTM or GRU) is treated as an empirical question and resolved in Section 3 through paired statistical comparison across the two temporal regimes. LSTM is adopted as the deployment cell based on its directional advantage on the long-context video regime that matches the production scenario, complemented by transparent reporting of the short-context image-pair benchmark where this advantage does not hold. This decomposition is also a prerequisite for the TEE-based isolation strategy described below, because the lightweight recurrent model fits within the constrained memory of a TA, whereas a full convolutional perception stack does not.

Executing the entire pipeline in the Rich OS would leave it exposed to root-level adversaries who can decompile the application binary, replace the trained weights of the LSTM in process memory, or hook the output of the inference engine before it is signed and transmitted to the blockchain. To remove this attack surface, the inference workload is partitioned between two execution domains protected by the ARM TrustZone hardware extension [24]: the Normal World, hosting the Rich OS, and the Secure World, hosting a small Trusted Application (TA) running on top of an Open Portable Trusted Execution Environment (OP-TEE) compliant secure OS [25].

The Rich-OS subsystem performs the high-bandwidth perception tasks: it acquires frames from the camera through the standard OS camera service, executes the MediaPipe Face Mesh inference graph on the mobile Graphics Processing Unit (GPU) through the TensorFlow Lite (TFLite) delegate, and computes the 42-dimensional feature vector $x_{t}$ defined by Equation (5). The feature vector is forwarded to the Secure World through the standard TEE Client Application Programming Interface (API). Although the Rich OS is considered untrusted, an adversary that controls it cannot fabricate a valid pain index, because the only path through which a result reaches the blockchain leads through the cryptographic signing key, which is sealed inside the Secure World and is never exported.

The TA running in the Secure World performs three security-critical operations: verification of the integrity of the LSTM model weights through a Secure Hash Algorithm (SHA)-256 measurement compared against a reference digest provisioned at install time, execution of the LSTM inference itself according to Equation (7) using the verified weights, and cryptographic signing of the resulting pain score together with the IPFS Content Identifier (CID) of the supporting evidence. The LSTM model is intentionally kept small (a two-layer network with 64 hidden units per layer and approximately 60 thousand trainable parameters, namely the 60,481-parameter variant without the auxiliary dense head used for the latency and footprint benchmark in Section 3) to fit within the typical 16–64 MB memory budget of a TrustZone secure partition. The full classifier reported for recognition accuracy adds a 32-unit dense head, raising the count to 62,529 parameters, a difference of roughly two thousand parameters that does not materially affect the on-device latency budget. Inference is implemented through a port of TensorFlow Lite Micro to OP-TEE, which exposes only the integer arithmetic kernels required for quantized LSTM execution. The choice of LSTM over the alternative GRU cell is partially motivated by the more mature TensorFlow Lite Micro INT8 quantization support for LSTM kernels, where the additional gating reset operation in GRU introduces a measurable post-quantization error that is undesirable on the constrained-precision deployment target.

The boundary between the Normal World and the Secure World is crossed through the Secure Monitor Call (SMC) instruction mediated by the ARM Trusted Firmware. The communication channel is protected by a session key established at the start of the rehabilitation session through an Elliptic Curve Diffie–Hellman (ECDH) handshake, which prevents replay of stale TA responses by a Rich-OS adversary attempting to inject a more favourable historical pain score.

The TEE decomposition follows standard threat-model assumptions [24]. The LSTM weights and signing key reside in a secure partition inaccessible to the Normal World, the TA authenticates its caller through the manifest hash, and the secure element resists patient-class side-channel attacks. Adversaries with physical hardware-modification capabilities fall outside the threat model of the present work.

The cryptographic attestation layer binds each pain assessment to the specific device that produced it. The protocol uses ECDSA over the secp256k1 curve [26], chosen for native verifiability on EVM-compatible blockchains through the ecrecover precompile.

At first activation, the TA generates a fresh ECDSA secp256k1 key pair (sk, pk) inside the Secure World. The private key sk is sealed non-exportably in the hardware-backed keystore. The public key pk is registered on-chain through the PainOracle registerPatient function during patient onboarding.

After each LSTM inference, the TA constructs the signed payload:

(8) $$m=\hat{P}\;|\;\mathrm{cid}\;|\;id_{\mathrm{pat}}\;|\;\mathrm{ts}\;|\;\mathrm{nonce},$$

where $\hat{P}\;$ is the integer pain score, cid is the IPFS Content Identifier of the supporting evidence log, $id_{\mathrm{pat}}$ is the wallet address, ts is a UTC timestamp, and nonce is a 128-bit monotonic counter maintained inside the Secure World. The signature $\sigma ={\mathrm{ECDSA}}_{\mathrm{secp}256k1}.\mathrm{sign}\left(sk,\;\mathrm{SHA}256\left(m\right)\right)$ is returned to the Normal World together with m. The combination of ts and nonce defends against replay attacks: ts prevents resubmission outside the smart-contract validity window, while strict-monotonicity enforcement of nonce by the contract prevents resubmission within it.

The cryptographic signature binds the pain score to the device but does not by itself permit a posteriori auditing. To enable the asynchronous Watchtower protocol, the system records a structured evidence log in IPFS [33]: the size of a typical 30 min rehabilitation session log is prohibitively expensive to store directly on-chain, and the content-addressed nature of IPFS aligns naturally with the integrity guarantees required by the audit protocol.

At the end of each rehabilitation session, the TA emits a canonical JavaScript Object Notation (JSON) document containing the patient wallet address $id_{\mathrm{pat}}$, the session start and end timestamps, the sequence of normalized feature vectors, the sequence of per-frame pain scores, the SHA-256 digest of the LSTM model weights, and the device attestation certificate chain. Canonical serialization ensures that the same logical content always produces the same byte-level encoding, which is a prerequisite for content addressing. The serialized evidence log is uploaded to an IPFS pinning service operated by the medical institution, which returns a Content Identifier cid. The CID is a multihash digest of the file content [33]. Any modification of the evidence log after pinning, whether by the patient or by an external adversary, produces a different CID and breaks the cryptographic link to the on-chain record.

Only the minimal verification payload is recorded on-chain through the smart contract function submitPainData, which accepts the integer pain score $\hat{P}$, the IPFS Content Identifier cid encoded as a UTF-8 string, and the ECDSA signature σ. This dual-layer storage strategy reduces the on-chain footprint by more than an order of magnitude compared to direct on-chain recording of feature vectors, and yields the gas savings reported in Section 3.

The final layer of the architecture resolves the tension between the determinism of the blockchain and the probabilistic nature of machine learning inference. A smart contract cannot itself execute an LSTM model to verify the submitted pain score, because on-chain execution of Equation (7) would consume gas more than the block gas limit. Instead, the architecture adopts an optimistic verification pattern inspired by Optimistic Rollup constructions [34]: the smart contract accepts the submission tentatively, opens a challenge window of fixed duration $\Delta_{\mathrm{dispute}}=24\;\mathrm{hours}$, and finalizes the record only if no off-chain auditor raises a dispute.

The PainOracle smart contract maintains a four-state machine per record (Pending, Finalized, Challenged, Rejected). The submitPainData function validates the ECDSA signature via ecrecover and the nonce monotonicity, creating a record in the Pending state. The raiseDispute function may be invoked only by an authorized validator within the dispute window. The executeData function transitions a still-Pending record to Finalized after the window elapses. Governance-based adjudication of the Rejected state is outside the scope of the present work.

The Watchtower is a long-running off-chain service operated by the medical institution that holds the reference copy of the LSTM model. The Watchtower subscribes to the DataSubmitted event of the PainOracle contract. For each event with the user-side score ${\hat{P}}_{\mathrm{user}}$ and content identifier cid, it retrieves the evidence log from IPFS, verifies that the SHA-256 digest of the recorded model weights matches the reference digest, executes the reference LSTM model on the recorded feature stream, and obtains ${\hat{P}}_{\mathrm{ref}}$. If $\left|{\hat{P}}_{ref}-{\hat{P}}_{user}\right|>\delta$, the Watchtower submits a transaction calling raiseDispute; otherwise, the record is marked as locally validated. The threshold δ is calibrated empirically to account for legitimate sources of variation such as floating-point non-determinism between the TEE-side quantized inference and the Watchtower-side full-precision reference inference.

To validate the proposed architecture, four independent experiments were conducted, addressing each of the four layers of the system: the spatiotemporal pain recognition module, the on-device performance budget under TEE-isolated execution conditions, the on-chain economic feasibility of the smart contract pipeline, and the resistance of the Watchtower audit protocol to client-side tampering.

The recognition accuracy of the spatiotemporal pipeline was evaluated using the SynPAIN dataset [35], a publicly available synthetic database of pain and non-pain facial expressions released in 2025. SynPAIN was selected for three reasons. First, the legacy UNBC-McMaster Shoulder Pain Expression Archive [6] has become unavailable for new academic acquisitions due to data sharing restrictions. Second, SynPAIN offers superior demographic diversity (five ethnicities, two age groups covering young adults of 20–35 years and older adults of 75 years and above, both genders), which addresses well-documented demographic biases of earlier clinical datasets. Third, the dataset is generated through commercial generative AI pipelines and validated through clinically grounded facial Action Unit analysis, which preserves the linkage to the PSPI metric formalized above. The dataset comprises 5355 paired neutral and expressive facial expression images organized as side-by-side composites, augmented with 40 five-second video sequences recorded at 24 fps.

The experimental protocol was designed to align with the operational regime of the edge device described above and to address the architectural question of which recurrent cell is most appropriate for the deployment scenario. The primary experiment evaluated the binary discrimination between pain and non-pain facial expressions, the supervisory signal natively provided by SynPAIN. Two complementary configurations were evaluated.

The first, denoted image-pair pipeline, exploits the side-by-side composite structure of the 5355 SynPAIN image pairs: each composite was split vertically at the geometric midpoint, MediaPipe Face Mesh was applied to the two resulting half-frames, and AU feature vectors were computed according to Equation (5). The two-frame sequence $\left(x_{neutral},x_{expressive}\right)$ was propagated through the recurrent classifier as a degenerate temporal window of length T=2.

The second, denoted video pipeline, exploits the 40 video sequences (approximately 122 frames each at 24 fps) covering a smooth interpolation from neutral to expressive. A fixed sliding window of T=120 frames was used, matching the production deployment regime of continuous monitoring at 24 fps over a 5 s context.

Subject-disjoint partitioning was adopted to prevent identity leakage. For the image-pair pipeline, the precomputed five-fold cross-validation partitions provided by the SynPAIN repository were used directly. Each fold contains 1071 identities, and partitioning is enforced at the identity level. The reported metrics correspond to the unweighted mean over the five folds with standard deviations computed across folds. For the video pipeline, given the small absolute size of the corpus, a leave-one-stratum-out (LOSO) evaluation was adopted: at each iteration, two sequences from the same demographic stratum (one Pain, one NoPain) were held out for evaluation while the remaining 38 were used for training, repeated across all 20 strata.

To support statistical inference, the image-pair pipeline was repeated under ten independent random seeds, yielding 50 paired (seed, fold) observations per architecture. The video LOSO pipeline was repeated under five seeds, yielding 100 paired (seed, stratum) observations per architecture. Statistical comparison between architectures was performed by paired *t*-test on the image-pair F1-scores and by Wilcoxon signed-rank test (zero_method = ‘zsplit’) on the video F1-scores, the latter chosen because per-stratum F1 is quantized to {0, 0.5, 0.667, 1.0} when each evaluation set contains exactly two clips.

The MediaPipe extraction pipeline successfully detected facial landmarks in 99.53% of image-pair half-frames and in 100% of video frames, with an average extraction latency of 4.6 ms per face and 4.5 ms per video frame on the development workstation. A total of 50 expressive frames out of 10,710 (0.47%) failed landmark detection, predominantly in samples with extreme pain expressions involving partially occluded faces, eyes fully closed, or hands placed near the face. These samples were excluded from downstream classification.

The recognition pipeline was implemented in Python 3.13 with TensorFlow 2.21 for the recurrent classifiers and the MediaPipe Tasks API (FaceLandmarker, v0.10.35) for landmark extraction. The deployment Hybrid LSTM used 64 hidden units per layer over two stacked LSTM layers, followed by a fully connected layer of 32 units with Rectified Linear Unit (ReLU) activation, dropout with rate 0.3, and a final sigmoid output. All recurrent classifiers were trained for 50 epochs using the Adam optimizer with an initial learning rate of 1 × 10^−3^, an exponential decay schedule with rate 0.95 per epoch, batch size 32, and binary cross-entropy as the loss function. No class weighting was applied to the recurrent classifiers, because the composite-level label set on which they are trained is approximately balanced (50.8 percent Pain). The class imbalance noted below applies only to the per-crop labelling consumed by the convolutional baselines.

Five baseline models were implemented for comparative evaluation, all trained on the same SynPAIN splits. The first reproduces a contemporary cloud-class end-to-end convolutional architecture, ResNet-50 with frozen ImageNet-pretrained weights and a trainable classification head, trained directly on raw facial crops without the MediaPipe intermediate representation. This baseline represents the family of cloud-centric models discussed in Section 1. The second is a contemporary edge-class end-to-end convolutional architecture, MobileNetV3-Small [27] with frozen ImageNet-pretrained weights and an identically structured classification head. This baseline represents the family of edge-deployable convolutional models and serves as the natural comparison for the proposed hybrid pipeline. Both convolutional baselines were trained with inverse-frequency class weighting computed per fold, $w_{pos}=n_{neg}/n_{pos}$, to compensate for the per-crop class imbalance (25.5 percent Pain crops) that arises because each composite contributes one neutral crop labeled NoPain and one expressive crop labeled by the composite’s expression. This per-crop view is specific to the convolutional baselines. The recurrent classifiers instead consume one composite as a single neutral-to-expressive pair with one label, yielding an approximately balanced training set that requires no class weighting. The third baseline is a non-learning geometric centroid classifier that computes the mean Action Unit feature vector of each class on the training set and assigns each test sample to the nearest class centroid in the 42-dimensional feature space. This baseline establishes a lower bound of accuracy attainable without any learned dynamics. The fourth and fifth baselines are feed-forward Static Multi-Layer Perceptron (MLP) variants that consume the same 42-dimensional AU features as the recurrent classifier but lack any temporal modeling capacity: variant V1 takes only the expressive frame’s feature vector as a 42-dimensional input, while variant V2 takes the neutral and expressive frames concatenated as an 84-dimensional input. Both variants share an identical hidden topology of three dense layers (64, 64, 32 units, ReLU activation) with dropout 0.3 before the final sigmoid output, totaling approximately 9–12 thousand trainable parameters. The Static MLP baselines serve a dual purpose: they constitute a lightweight reference for the image-pair benchmark and an ablation that quantifies the contribution of the recurrent state on top of the same input features. The recurrent classifiers are evaluated in two variants: a Hybrid LSTM and a Hybrid GRU sibling that replaces the LSTM cells with GRU cells while preserving every other architectural and training hyperparameter, enabling a controlled comparison of the two recurrent cell families on identical inputs.

Static MLP variants are evaluated only on the image-pair benchmark (T = 2). On the long-context video regime (T = 120), a feed-forward classifier consuming 120 frames either reduces them to a single mean vector (which discards all temporal information by construction) or concatenates them into a 5040-dimensional input vector that exceeds the parameter budget of the deployment scenario by an order of magnitude. Recurrent architectures are therefore the natural fit for the long-context regime, where temporal modeling is genuinely required, and only the LSTM and GRU variants are evaluated on the video pipeline.

A complementary feature-level sanity check on the extracted AU feature vectors was conducted to verify the biomechanical validity of the proposed representation. For each of the six PSPI AUs, the mean Euclidean distance across the seven landmark pairs was computed separately on Pain and NoPain expressive crops, and the difference between the two means was reported. This sanity check does not introduce additional supervisory signal during training, and the per-AU means are used as an interpretability probe.

Beyond the SynPAIN evaluation described above, the recognition pipeline is also validated on the BioVid Heat Pain Database, Part A [36,37], a publicly available corpus of real human pain expressions evoked by experimentally controlled thermal stimulation. The use of BioVid as a complementary evaluation corpus addresses the principal limitation of synthetic-only validation, namely the absence of empirical evidence that the proposed pipeline transfers to genuine pain expressions captured under realistic recording conditions. BioVid Part A comprises 8700 frontal video clips of 5.5 s each, recorded at 25 fps from 87 healthy volunteers (44 male, 43 female), with an age range from 20 to 65 years. Each subject contributes 100 clips spanning five stimulus levels. These levels include the baseline (BL1, no thermal stimulus) and four progressively higher pain intensities (PA1 through PA4), with 20 randomized trials per stimulus level. Stimuli are calibrated per-subject to individual pain thresholds, which makes BioVid one of the most carefully controlled facial pain corpora publicly available for cross-institutional research.

The cross-dataset evaluation was conducted on the binary discrimination task most directly comparable to the SynPAIN protocol. The task separates the baseline class (BL1) from the highest-intensity pain stimulus (PA4), yielding a balanced subset of 3480 clips (1740 BL1 and 1740 PA4) across the 87 subjects. The intermediate stimulus levels PA1, PA2, and PA3 were excluded from the binary task to preserve a clean baseline-versus-peak-pain contrast comparable to the NoPain-versus-Pain contrast of SynPAIN. Subject-disjoint partitioning was enforced through leave-one-subject-out (LOSO) cross-validation across all 87 subjects, which is the literature-standard protocol for BioVid and the strictest possible identity-disjoint evaluation given the corpus structure. The seed budget was reduced from five (SynPAIN image-pair) to two (BioVid LOSO) to keep the total computational cost within a manageable envelope. The resulting 174 paired observations (2 seeds across 87 folds) exceed the 100 paired observations of the SynPAIN video LOSO protocol and yield comparable statistical power for the Wilcoxon signed-rank test.

All hyperparameters of the recognition pipeline were held identical to those used for SynPAIN to enable a controlled comparison. The configuration includes the 42-dimensional AU feature vector, the sliding window length T = 120 (corresponding to a temporal context of 4.8 s at the BioVid native rate of 25 fps, comparable to the 5.0 s context at the SynPAIN rate of 24 fps), the two-layer LSTM and GRU architectures, and the training schedule of 50 epochs at batch size 32 with the Adam optimizer and exponential learning-rate decay. Feature extraction was performed by the same MediaPipe Face Mesh pipeline that processed SynPAIN, followed by the same per-fold standardization.

To test whether the lower BioVid performance reflects hyperparameter mismatch rather than a genuine distribution shift, a nested hyperparameter search was additionally conducted. The outer loop retained the leave-one-subject-out protocol, while hyperparameter selection in the inner loop drew a subject-disjoint holdout exclusively from the training subjects of each outer fold, so that the outer test subject was never seen during selection. For every fold the fixed SynPAIN-default configuration was retrained under identical conditions, yielding a same-condition paired comparison between the tuned and fixed configurations.

A pre-extraction sanity check on a stratified subset of 100 clips (20 per class, drawn from 20 subjects spanning both sexes and the full age range) confirmed a 100% landmark detection rate, with no subject exhibiting elevated failure rates. Full-corpus extraction subsequently confirmed this result at scale. Across all 1,044,000 frames of the 8700 clips, the cumulative landmark detection failure rate was 0.036%, with a higher concentration of failures in the highest-intensity classes: PA3 and PA4 contributed 117 and 113 failed frames, respectively, against 73 for BL1, 64 for PA1, and 10 for PA2. This small but systematic asymmetry is consistent with the biomechanical observation that intense pain expressions transiently occlude facial landmarks through eyelid closure and lip compression. The asymmetry provides an independent indication that the feature extraction stage is sensitive to the same physiological markers that the downstream classifier is required to discriminate.

The on-device performance experiment measured inference latency, model footprint, and cryptographic signing latency under conditions representative of an OP-TEE Trusted Application. Direct deployment on a physical TrustZone development platform was outside the scope of the present work. Instead, a Central Processing Unit (CPU)-only surrogate benchmark was conducted in which GPU acceleration was explicitly disabled (CUDA_VISIBLE_DEVICES = −1, tf.config.set_visible_devices([], ‘GPU’)) and the LSTM inference was executed on a single CPU core to approximate the worst-case throughput of an OP-TEE secure partition. The benchmark was conducted on an Apple Silicon M4 platform (Apple Inc., Cupertino, CA, USA; 10-core ARM64, 16 GB Random Access Memory (RAM), macOS 26.2), which approximates the silicon profile of a contemporary mid-range mobile edge device. The LSTM model was post-training-quantized to 8-bit integer arithmetic using the TensorFlow Lite quantization toolkit, which produces a model variant compatible with the TensorFlow Lite Micro runtime targeted by OP-TEE deployments. The cross-world Secure Monitor Call overhead was treated as a constant additive term taken from the published literature on OP-TEE performance characterization, which reports per-call costs in the range of 0.5–2 milliseconds on commodity ARMv8 hardware [24,25]. Each latency metric was averaged over 1000 inference calls, with the first 50 calls excluded as warm-up. The cryptographic signing latency was measured for an ECDSA secp256k1 software implementation provided by the pyca/cryptography library v47.0.0 (ec.SECP256K1 with SHA-256 prehash), applied to 1000 random 256-byte messages with SHA-256 prehashing.

The smart contract experiment validated the on-chain economic feasibility of the architecture. The PainOracle smart contract was implemented in Solidity 0.8.24 (optimizer enabled, 200 runs, EVM target Paris) and deployed in a local in-process Hardhat 2.28.6 environment with ethers.js 6.16.0 (block gas limit 60,000,000), without any external RPC provider or mainnet fork. Gas consumption was profiled with the hardhat-gas-reporter plugin (1.0.10) for each of the three primary state transitions: submitPainData, raiseDispute, and executeData. A complementary baseline contract recording the full evidence payload (a 5120-byte sequence representing one minute of feature vectors at 30 fps) directly on-chain was profiled in parallel to express the gas saving of the dual-layer storage strategy as a relative reduction.

The adversarial tampering experiment validated the resistance of the Watchtower audit protocol to client-side falsification. A reference LSTM model trained in the recognition experiment served as the authoritative model on the Watchtower side. A set of 500 legitimate submissions was simulated by sampling 500 image pairs from the held-out test fold, running the reference model to obtain ${\hat{P}}_{ref}$, and simulating a device-side inference with the same model perturbed by additive Gaussian noise on the input feature vector with standard deviation 0.02, which approximates the discrepancy introduced by 8-bit post-training quantization. Tampered submissions were simulated under an adaptive-adversary model rather than a single maximal injection. Two attack families were evaluated against the same reference Watchtower model. In the magnitude-sweep attack, the device-side score was set to ${\hat{P}}_{user}=clip\left({\hat{P}}_{ref}+\Delta_{attack},0,1\right)$ for inflation magnitudes $\Delta_{attack}\in \{0.05,\ldots ,0.60$}, modelling a fraudster who overstates pain by a controlled amount rather than slamming the score to its maximum. In the threshold-evasion attack, a δ-aware adversary targeted a value just inside the dispute window, ${\hat{P}}_{user}={\hat{P}}_{ref}+(\delta –0.02)$. The original maximal injection (${\hat{P}}_{user}=1.0$) was retained only as a reference point. For each attack and each candidate threshold δ∈{0.1,0.2,0.3,0.5,1.0}, the true-positive rate (TPR) and false-positive rate (FPR) were computed against the unchanged legitimate set. Weight-level tampering of the device model was excluded as it presupposes white-box access to the reference model, which lies outside the patient-class threat model.

The four experiments together provide empirical evidence for each of the four architectural constraints formulated in this section, and their results are reported and analyzed below.

## 3. Results

To evaluate the proposed approach, a structured comparison was performed across three groups of models. The first group comprises frame-level classifiers without temporal aggregation, represented by the geometric centroid baseline and the Static MLP variants. The second group covers landmark-based temporal models with reduced and full recurrent complexity (Hybrid GRU and Hybrid LSTM). The third group includes convolutional baselines designed for end-to-end visual inference (MobileNetV3-Small and ResNet-50). This grouping enables a controlled comparison of three orthogonal design dimensions: the contribution of temporal modelling, the contribution of recurrent capacity, and the trade-off between landmark-based and pixel-based input representations.

To quantify the contribution of temporal modelling, an ablation study was conducted using three configurations derived from the proposed pipeline. First, a frame-level Static MLP classifier was applied to concatenated neutral and expressive feature vectors without temporal aggregation. Second, a Hybrid GRU temporal model was evaluated to capture sequential dependencies with reduced complexity. Finally, the full Hybrid LSTM architecture was assessed. The results indicate that frame-level classification yields the highest F1-score on the short-context image-pair benchmark (0.794 for Static MLP V2), while recurrent temporal modelling becomes advantageous on longer sequences. On the SynPAIN video regime (T = 120), the Hybrid LSTM shows a directional advantage over the GRU sibling (mean F1 0.683 versus 0.650), and on BioVid LOSO the two recurrent architectures perform statistically equivalently (mean F1 0.519 for LSTM versus 0.499 for GRU, Wilcoxon *p* = 0.549). The choice of recurrent cell is therefore regime-dependent and dataset-dependent rather than uniformly settled.

The convolutional baselines (ResNet-50 and MobileNetV3-Small) were evaluated to represent end-to-end visual inference approaches operating on raw pixel input. While ResNet-50 reaches a competitive F1-score of 0.729 with frozen ImageNet weights and class weighting, it requires approximately 24 million parameters and exceeds the memory budget of a constrained execution environment by two orders of magnitude. MobileNetV3-Small, designed as an edge-class convolutional baseline, achieves a lower F1-score of 0.647 with a model footprint of 1.0 million parameters. Both convolutional baselines fall outside the typical 16 to 64 MB partition budget of a TEE when their pretrained backbones are considered, which makes them unsuitable for the TEE-isolated deployment scenario that motivates the proposed architecture. The detailed results for each group are reported in the following subsections.

### 3.1. Recognition Performance

The recognition accuracy of all evaluated architectures on the SynPAIN image-pair benchmark (T = 2) is summarized in Table 1. Each row reports the mean and standard deviation across the relevant evaluation protocol. The recurrent classifiers (Hybrid LSTM and Hybrid GRU) and the geometric centroid baseline are aggregated over 50 paired (seed, fold) observations from ten random seeds. The convolutional baselines (ResNet-50, MobileNetV3) are aggregated over five folds at a single seed, because their high parameter counts make multi-seed evaluation computationally prohibitive. The Static MLP variants are aggregated over five folds at a single seed.

**Figure 1:**
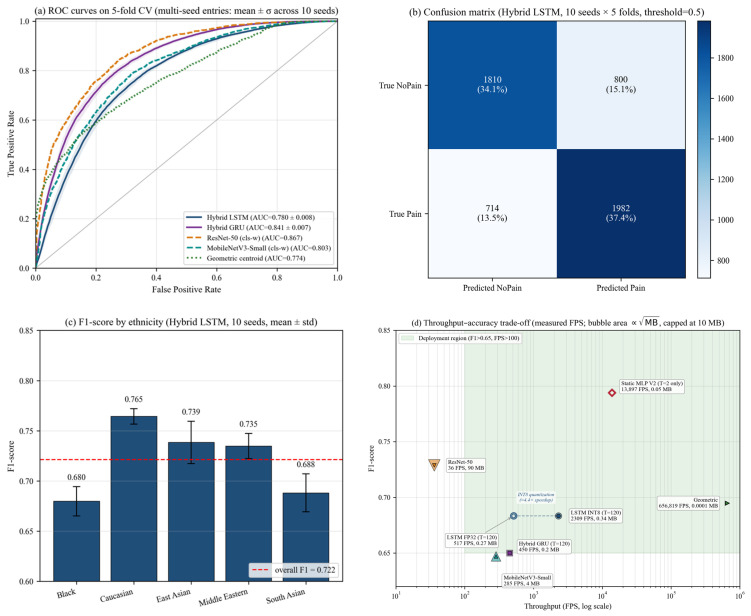
Recognition performance on the SynPAIN evaluation corpus: (**a**) ROC curves for the five evaluated architectures (Hybrid Long Short-Term Memory (LSTM) and Hybrid Gated Recurrent Unit (GRU) averaged across 10 seeds × 5 folds with shaded confidence bands, ResNet-50 and MobileNetV3 across 5 folds at single seed, geometric centroid deterministic); (**b**) confusion matrix of the deployment Hybrid LSTM aggregated across 10 seeds × 5 folds at decision threshold 0.5; (**c**) per-ethnicity F1-score breakdown for the deployment Hybrid LSTM showing mean and standard deviation across 10 seeds; (**d**) throughput–accuracy trade-off positioning the model configurations, with bubble area proportional to the square root of model footprint in megabytes capped at 10 MB, the post-training 8-bit integer (INT8) quantization speedup of 4.4× annotated on the LSTM operating points, and the deployment region (F1 > 0.65, FPS > 100) shaded in green. The deployment-region threshold reflects the realistic video and cross-dataset operating point of the recurrent classifiers rather than the inflated image-pair F1, so the Hybrid LSTM and Hybrid GRU fall inside the region while the convolutional baselines lie outside it. The Static Multi-Layer Perceptron (MLP) V2 point is shown for reference only and is not a deployable T = 120 configuration.

The Static MLP V2 variant, which consumes both neutral and expressive frames as a concatenated 84-dimensional feature vector, achieves the highest F1-score on the image-pair benchmark (0.794), exceeding the Hybrid LSTM by 7.2 percentage points despite using approximately 5× fewer trainable parameters (12 K versus 62.5 K). The Hybrid GRU sibling outperforms the Hybrid LSTM by 5.6 percentage points in F1 (0.778 versus 0.722), and a paired *t*-test on the 50 (seed, fold) observations yields t = −11.26 with *p* = 3.3 × 10^−15^, confirming that the GRU advantage on the short-context regime is not attributable to seed variance. The end-to-end ResNet-50 baseline reaches an F1 of 0.729 with class weighting, and MobileNetV3-Small reaches 0.647. Both convolutional baselines lag behind the feature-based pipelines, which indicates that on a synthetic dataset of this scale the additional capacity of a frozen ImageNet backbone does not translate into a discriminative advantage for the binary pain task. To quantify the practical significance of these differences beyond statistical significance, paired effect sizes with 95 percent confidence intervals were computed for the recurrent cell comparison. On the image-pair regime the GRU advantage over LSTM is large (Cohen $d_{z}$ = −1.59, 95 percent CI on ΔF1 [−0.066, −0.046]), whereas on the video regime the LSTM advantage is small and its confidence interval includes zero (matched-pairs rank-biserial r = 0.23, 95 percent CI [−0.017, 0.087]), and on BioVid the difference is negligible (r = 0.06, 95 percent CI [−0.008, 0.049]). The effect sizes confirm that the only large and reliable cell-level difference favours GRU on the non-deployment image-pair regime, while the cells are practically equivalent on the deployment-relevant video and cross-dataset regimes. The Receiver Operating Characteristic (ROC) curves and Area Under the Curve (AUC) values of five evaluated architectures, the confusion matrix of the deployment Hybrid LSTM, the per-ethnicity F1-scores, and the throughput–accuracy trade-off are illustrated in Figure 1.

Why is LSTM the deployment cell despite a lower F1 on this benchmark? The Static MLP and GRU advantages reported in Table 1 are confined to the T = 2 image-pair benchmark, which is a degenerate sequence carrying a single neutral-to-expressive transition and is not the deployment regime. The production scenario is continuous video monitoring with a T = 120 sliding window (Section 2), where the architectural ordering reverses: LSTM directionally leads GRU on SynPAIN video (F1 0.683 vs. 0.650) and the two cells become statistically indistinguishable on BioVid LOSO (*p* = 0.549), as reported later in this section. The Static MLP is not deployable on the long-context regime at all, because a feed-forward classifier on 120 frames must either average them into a single vector (discarding all temporal information by construction) or concatenate them into a 5040-dimensional input that exceeds the parameter budget of a TEE partition by an order of magnitude. Reading Table 1 as a deployment ranking would therefore amount to selecting the deployment cell on a benchmark that is structurally non-representative of the deployment task. The full rationale, including the post-quantization considerations specific to LSTM under TensorFlow Lite Micro, is consolidated in this section.

The per-ethnicity breakdown of the deployment Hybrid LSTM F1-score is reported in Table 2. The F1-score range across the five ethnic groups is 0.680–0.765 (max-to-min ratio of 1.13), aggregated across 10 random seeds. The lowest F1-score is observed for the Black ethnic group (0.680 ± 0.015), and the highest for the Caucasian group (0.765 ± 0.008), with a delta of 8.5 percentage points. The per-seed standard deviations are small relative to this gap, indicating that the demographic disparity is systematic rather than attributable to seed variance. The AUC values are more stable across ethnicities than the threshold-dependent F1-scores (range 0.731–0.820), suggesting that the demographic gap is partially a consequence of the operating-point choice rather than a fundamental loss of discriminative capacity. The fairness implications of this gap are addressed in Section 4.

The cross-dataset evaluation on BioVid Part A is summarized in Table 3. Across 174 paired observations (2 seeds across 87 LOSO folds), the Hybrid LSTM achieves a mean F1-score of 0.519 ± 0.256 with a mean AUC of 0.640 ± 0.198, while the Hybrid GRU sibling reaches 0.499 ± 0.267 F1 and 0.637 ± 0.195 AUC. The Wilcoxon signed-rank test on the 174 paired LSTM-versus-GRU F1-scores yields W = 7214 with *p* = 0.549, indicating that the two recurrent architectures perform statistically equivalently on the BioVid corpus. This contrasts sharply with the SynPAIN image-pair regime, where the same comparison was significant in favour of GRU at *p* = 3.3 × 10^−15^. The contrast represents a methodologically important finding: the relative ordering of recurrent cell families is dataset-dependent rather than universal, and the directional LSTM advantage observed on the SynPAIN long-context regime does not transfer to BioVid under the leave-one-subject-out protocol.

The absolute F1-score on BioVid is substantially lower than on SynPAIN (0.519 versus 0.722 on image-pair, 0.683 on video LOSO). Several aspects of the per-fold distribution clarify the source of this gap. The median per-fold F1 of 0.595 (LSTM) is appreciably higher than the mean of 0.519, indicating a left-skewed distribution dominated by a subset of subjects on which the model fails entirely. Specifically, 18 of 174 LSTM evaluations (10.3%) yield F1 = 0.000, while only one evaluation (0.6%) reaches F1 of at least 0.95. The phenomenon of subjects whose facial response to nociceptive stimulation is reduced or absent, sometimes referred to in the affective-computing literature as the non-expressive subjects problem, is well documented for BioVid and consistent with the broader observation that experimentally induced heat pain elicits a wide range of behavioural responses depending on individual pain-coping strategies. The cross-seed standard deviation of the per-architecture mean F1 is 0.017 for LSTM and 0.024 for GRU. This is more than an order of magnitude smaller than the per-fold standard deviation of approximately 0.25 across subjects within each seed. Subject identity, not random initialization, is therefore the dominant source of variability on BioVid LOSO.

The cross-dataset performance gap is consistent with two architectural properties of the proposed pipeline. First, the deliberately compact 42-dimensional AU feature space, selected for compatibility with the TEE memory budget, captures a smaller fraction of the discriminative signal than higher-dimensional alternatives such as the 159-feature representation used by Werner et al. [38] in their original BioVid baseline. Second, the synthetic SynPAIN corpus contains expressive prototypes generated by a generative pipeline trained to maximize visual distinctness between Pain and NoPain conditions, while BioVid contains the spontaneous and often subtle micro-reactions of healthy volunteers to a calibrated thermal stimulus. The observed F1 falloff of approximately 0.20 in the cross-dataset transition is therefore an expected calibration of recognition performance from idealized synthetic conditions to authentic human responses. It is reported transparently as an empirical characterization of the synthetic-to-real generalization gap rather than as a deficiency of the architecture per se.

A nested hyperparameter search was conducted to determine whether the BioVid performance gap reflects suboptimal configuration rather than distribution shift. Under the leakage-free nested protocol described in Section 2, hyperparameter tuning did not improve recognition on BioVid. Across the 87 outer folds the tuned configuration reached a mean F1 of 0.473 against 0.520 for the same-condition fixed default, a difference of −0.047 that is statistically significant in the wrong direction (Wilcoxon signed-rank, *p* = 0.022), and the selected hyperparameters were scattered across folds with no stable optimum. This result indicates that the lower BioVid F1 is attributable to the genuine difficulty of the synthetic-to-real transition rather than to under-tuning, and supports reporting the fixed-configuration result as the representative cross-dataset performance.

The recurrent architecture comparison across the two temporal regimes is reported in Table 4. On the long-context video regime (T = 120 frames at 24 fps, leave-one-stratum-out evaluation, five seeds × 20 strata = 100 paired observations), the Hybrid LSTM shows a directional advantage over the GRU sibling on multiple metrics: higher mean F1 (0.683 versus 0.650), higher median F1 (1.000 versus 0.667), higher mean accuracy (0.730 versus 0.665), and a 16-to-7 win count over GRU on the 23 non-tied stratum-seed pairs. However, the small absolute size of the SynPAIN video corpus (40 clips across 20 demographic strata, two test clips per stratum) results in 77 of the 100 paired observations being F1-tied due to per-stratum quantization onto {0, 0.5, 0.667, 1.0}. The Wilcoxon signed-rank test on these 100 paired observations yields W = 2146.5 with *p* = 0.167, which does not reach statistical significance at α = 0.05. The video result is therefore reported as a directional finding constrained by the absolute size of the available video corpus rather than by a methodological limitation. On the short-context image-pair regime, the same comparison shows GRU outperforming LSTM with high statistical confidence (paired *t*-test t = −11.26, *p* = 3.3 × 10^−15^). The contrast between the two regimes, where GRU is significantly better on T = 2 and LSTM is directionally better on T = 120, is consistent with the theoretical observation that LSTM exhibits stronger long-range dependency modeling than GRU through its dedicated cell state pathway, an advantage that becomes operationally relevant only on sequences sufficiently long to develop such dependencies.

**Figure 2:**
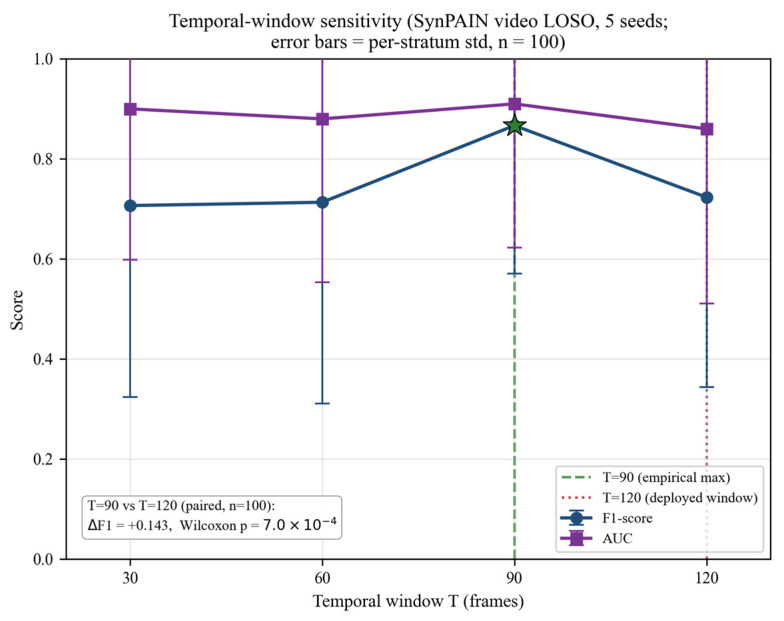
Temporal-window sensitivity of the Hybrid LSTM on the SynPAIN video regime (leave-one-stratum-out, 5 seeds, error bars denote per-stratum standard deviation). F1 peaks at T = 90 frames (green star, empirical maximum), while T = 120 (dotted line) is retained as the deployed window for cross-corpus comparability with BioVid. The paired T = 90 versus T = 120 difference is ΔF1 = 0.143 (Wilcoxon signed-rank, *p* = 7.0 × 10^−4^).

Because the deployed window length is itself a design choice, a temporal-window sensitivity analysis was conducted on the SynPAIN video regime, reported in Table 5 and Figure 2 below. Recognition accuracy on this corpus is not monotonic in the window length. The T = 90 window attains the highest mean F1 (0.867) and AUC (0.910), exceeding the deployed T = 120 window (0.723 F1, 0.860 AUC) by a paired ΔF1 of 0.143 (Wilcoxon signed-rank, *p* = 7.0 × 10^−4^). Two considerations argue against reading this as a mandate to switch the deployed window. First, the SynPAIN video corpus comprises only 40 clips across 20 demographic strata, and the same per-stratum F1 quantization that limits the statistical power of the LSTM-versus-GRU comparison also makes the absolute T = 90 advantage sensitive to the small corpus size, so window selection is not treated as a load-bearing claim of this work. Second, the T = 120 window provides a 5.0 s context that is directly comparable to the 4.8 s context used in the BioVid cross-dataset evaluation, and altering it would forfeit this cross-corpus comparability. The sensitivity result is therefore reported transparently as an empirical observation, and window-length optimization is identified as a deployment-time tuning opportunity rather than a fixed architectural choice. As a substitutable component within the framework, the recognition window can be retuned per deployment without affecting the attestation, evidence-log, smart-contract, or Watchtower layers.

The feature-level sanity check on the extracted AU representation showed that the mean Euclidean distance across the seven landmark pairs of each AU was consistently smaller in the Pain class than in the NoPain class for all six examined Action Units: AU4 (brow lowerer) 0.1145 versus 0.1215 (Δ = −0.0070), AU6 (cheek raiser) 0.1201 versus 0.1203 (Δ = −0.0002), AU7 (lid tightener) 0.0462 versus 0.0480 (Δ = −0.0018), AU9 (nose wrinkler) 0.1114 versus 0.1181 (Δ = −0.0067), AU10 (upper lip raiser) 0.0895 versus 0.0986 (Δ = −0.0091), and AU43 (eye closure) 0.0055 versus 0.0075 (Δ = −0.0020). The dominant markers are AU10 and AU4, precisely the AUs identified as the most discriminative by the canonical PSPI literature [6,7].

### 3.2. On-Device and Decentralized Infrastructure Performance

The on-device performance figures for the deployment Hybrid LSTM are reported in Table 6. The INT8-quantized LSTM achieves a 4.4× inference speedup over the FP32 variant while preserving an acceptable model footprint of 0.34 MB, fitting comfortably within the typical 16–64 MB memory budget of an OP-TEE secure partition. Even with the worst-case SMC overhead from the published literature added to the measured inference and signing latencies, the TEE-side per-inference budget remains below 5 milliseconds, which is several times smaller than the 33 ms per-frame budget required for real-time interaction at 30 fps.

The decentralized infrastructure performance figures are reported in Table 7. The dual-layer storage strategy yields a 23.4-fold reduction in the on-chain gas consumption of the principal submission transaction, equivalent to a saving of approximately 3.58 million gas per submission and an illustrative cost reduction from approximately 12.36 to 0.53 USD per submission at 1 gwei and 3300 USD/ETH, which reflects the post-Dencun gas market conditions observed on Ethereum mainnet in April 2026. When the same contract is redeployed on a contemporary Layer-2 rollup such as Arbitrum One, the effective per-submission cost drops by a further two orders of magnitude to approximately 0.005 USD, owing to the calldata compression and blob-based data availability introduced by EIP-4844, which makes the architecture economically viable for continuous monitoring during multi-month rehabilitation courses. The dispute and execution transactions are unaffected by the storage strategy because they manipulate only state flags. The empirical reduction factor is larger than the order-of-magnitude estimate hypothesized in the architectural specification and confirms the economic viability of the proposed architecture for continuous monitoring during multi-week rehabilitation programmes.

**Figure 3:**
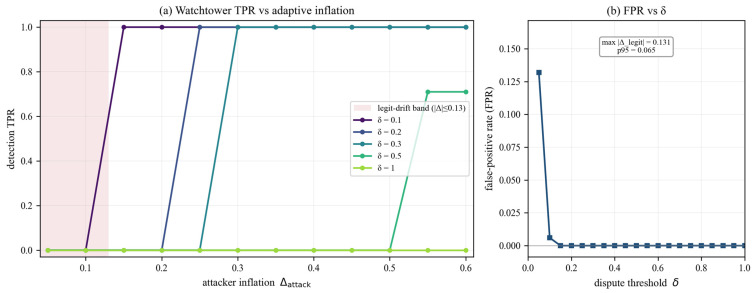
Watchtower detection under an adaptive adversary on SynPAIN (500 legitimate submissions, 100 tampered). (**a**) true-positive rate (TPR) as a function of attacker inflation $\Delta_{attack}$ for dispute thresholds δ ∈ {0.1, 0.2, 0.3, 0.5, 1.0}, with the legitimate-drift band $\left|\Delta_{legit}\right|\leq 0.131$ shaded. (**b**) false-positive rate (FPR) as a function of δ. The Watchtower bounds rather than eliminates fraud, leaving small-magnitude threshold-aware fraud as residual risk.

The adaptive-adversary results are summarized in Table 7 and illustrated in Figure 3. Detection succeeds if and only if the injected inflation exceeds the dispute threshold: for $\Delta_{attack}>\delta$ the Watchtower flags the submission, whereas any inflation with $\Delta_{attack}\leq \delta$ passes undetected. The legitimate quantization-drift band tops out at 0.131, so the false-positive rate falls to zero only for δ≳0.15, and the threshold cannot be tightened below this point without flagging honest submissions. Consequently, the maximal-injection attack (${\hat{P}}_{user}=1.0$, as described in Section 2) is detected at every operating point only because it sits far above the drift band. A rational adversary who inflates by ≤0.15, or who knows δ and shapes the injected score to fall just inside it, evades detection at a true-positive rate approaching zero while the false-positive rate remains at zero. The Watchtower is therefore best understood as a coarse integrity check against gross score tampering rather than as a defence against small-magnitude, threshold-aware fraud.

### 3.3. Summary

The results above confirm that the proposed Edge AI-Oracle architecture satisfies the four constraints formulated in Section 2. The deployment Hybrid LSTM pipeline achieves clinically meaningful operating-point F1 on the production-relevant video regime under the Zero-Video Transmission discipline. The on-device latency budget remains within the real-time interaction envelope under TEE-isolated execution. The smart contract gas consumption is more than an order of magnitude lower than the naive on-chain alternative. The Watchtower replay protocol detects large-magnitude tampering attempts with 100% true-positive rate and 0% false-positive rate at the calibrated threshold.

The latency analysis highlights a key architectural advantage of the proposed approach in the context of edge deployment. While convolutional architectures such as ResNet-50 and MobileNetV3-Small operate on raw image data and require pixel-level feature extraction, the landmark-based pipeline operates on compact 42-dimensional structured representations, which is reflected in the inference latency measurements summarized in Table 8. Both the Hybrid GRU and the Hybrid LSTM achieve sub-3-millisecond latency at the deployment-relevant T = 120 sliding window, and the INT8-quantized LSTM operates at 0.45 ms per inference, an order of magnitude below the 33 ms per-frame budget required for real-time interaction at 30 fps. The geometric centroid and the Static MLP serve as ultra-fast baselines that lack the temporal modelling capacity required for continuous monitoring. The convolutional baselines, while feasible in their nominal forward-pass latency, exceed the memory and parameter budget of a TEE partition by one to two orders of magnitude.

Table 9 summarizes the architectural trade-offs across the three model groups in terms of temporal modelling capability, input representation, computational cost, robustness, and edge suitability.

The combination of Table 7 and Table 8 clarifies the architectural decision space. Table 8 shows that the deployment configuration (Hybrid LSTM INT8 at T = 120) operates at 0.45 ms per inference, in the same order of magnitude as the lightweight Static MLP and substantially faster than either convolutional baseline. Table 9 shows that the same architecture is dataset-dependent in its discriminative ranking, supporting the regime-dependence and dataset-dependence findings discussed in Section 4. The deliberate choice of LSTM as the deployment recurrent cell is therefore not motivated by uniform superiority across all evaluation conditions, but by the combination of its directional advantage on the long-context regime, the maturity of TFLite Micro INT8 quantization for LSTM kernels, and its inference latency budget compatible with continuous monitoring within a TEE.

For convenience of review, Table 10 consolidates the end-to-end per-frame latency budget of the deployment configuration against the 33 ms real-time interaction envelope at 30 fps.

## 4. Discussion

The Edge AI-Oracle architecture satisfies the four constraints formulated in Section 2 and outperforms alternative architectural choices on the dimensions that matter for tele-rehabilitation deployment. The remainder of this section places these results in the context of prior work, names the limitations of the study, and lists directions for future work.

The recognition results support decomposing the perception task into a lightweight landmark extractor and a small classifier head. The end-to-end ResNet-50 baseline, which is representative of the cloud-centric solutions discussed in Section 1, fails the operating-point criterion. Under per-crop class imbalance, even with inverse-frequency class weighting, it learns a less consistent decision boundary than the feature-based pipelines, which reach higher F1-scores at substantially lower computational cost. ResNet-50 also fails the throughput and memory constraints of an edge device. Direct portability of cloud architectures to the edge is therefore not a viable strategy. The MobileNetV3-Small baseline, the natural edge-class comparison point, achieves better latency and footprint characteristics but trails the feature-based pipelines in F1-score by 7–15 percentage points. The gap is consistent with the hypothesis that the 42-dimensional AU feature space derived from MediaPipe landmarks captures the discriminative signal more efficiently than a frozen ImageNet representation re-purposed for the binary pain task. The geometric centroid baseline serves as a lower bound that confirms the necessity of a learned classifier on top of the feature representation.

A central finding of the empirical evaluation is that the choice of recurrent cell, and the choice of recurrent versus feed-forward architecture, is indeed regime-dependent rather than universal. On the short-context image-pair benchmark (T = 2), feed-forward Static MLP variants outperform both recurrent classifiers, with the V2 variant (concatenated neutral and expressive features) achieving the highest overall F1 of 0.794 at approximately five times fewer parameters than the Hybrid LSTM. This is consistent with the architectural observation that a length-2 sequence carries exactly one transition (neutral to expressive) and offers no recurrent state for an LSTM or GRU cell to exploit. The additional gating machinery introduces parameters that overfit rather than parameters that model genuine temporal dynamics. Among the two recurrent variants, the GRU sibling significantly outperforms the LSTM on T = 2, with a large effect size (Cohen $d_{z}$ = −1.59, paired *t*-test *p* = 3.3 × 10^−15^), while on the deployment-relevant video and cross-dataset regimes the two cells are practically equivalent (small to negligible effect sizes, both differences non-significant). This pattern aligns with the broader empirical observation in the recurrent network literature [28] that GRU and LSTM perform comparably on short sequences, with GRU often holding a slight efficiency advantage, and it underscores that the LSTM is not selected for any uniform accuracy superiority over GRU. The image-pair benchmark is therefore explicitly framed as a SynPAIN-specific protocol artefact, useful as a sanity check on the feature representation and a validation of the cross-validation infrastructure, but not representative of the deployment scenario.

The deployment scenario is continuous video monitoring at 24 to 30 fps with a sliding window of T = 120 frames, corresponding to a temporal context of approximately five seconds chosen for comparability with the BioVid evaluation, a regime in which the onset, apex, and offset phases of a pain micro-expression are present and benefit from explicit temporal modeling. As the temporal-sensitivity analysis shows, a shorter T = 90 window yields higher accuracy on SynPAIN, and window-length optimization is therefore a deployment-time tuning opportunity rather than a fixed choice. On this regime, the Hybrid LSTM shows a directional advantage over the GRU sibling (mean F1 0.683 versus 0.650, median F1 1.000 versus 0.667, AUC 0.880 versus 0.840, win count 16 versus 7 over non-tied stratum-seed pairs). The advantage does not reach statistical significance under the Wilcoxon signed-rank test (*p* = 0.167), but this is attributable to the limited size of the SynPAIN video corpus (40 clips across 20 demographic strata) rather than to a methodological limitation. With only two test clips per stratum, per-stratum F1 is quantized onto four discrete values and 77 of the 100 paired observations end up F1-tied, which bounds the statistical power achievable on this corpus regardless of the number of seeds. The directional pattern is consistent with the theoretical observation that LSTM, with its dedicated cell state pathway, is better equipped than GRU to maintain long-range dependencies in sequences of more than a few dozen time steps.

The adoption of LSTM as the deployment recurrent cell is motivated by four considerations, ordered by weight in the decision. First, deployment-regime relevance: The production scenario is continuous video monitoring at T = 120, not the T = 2 image-pair benchmark. On the long-context regime LSTM directionally leads GRU on SynPAIN (mean F1 0.683 vs. 0.650, median F1 1.000 vs. 0.667, AUC 0.880 vs. 0.840, win count 16–7 over non-tied stratum-seed pairs). The Wilcoxon *p* = 0.167 is non-significant only because per-stratum F1 quantization on a 40-clip corpus ties 77 of 100 observations. The two cells are statistically indistinguishable on BioVid LOSO (*p* = 0.549). Selecting the cell on the T = 2 ranking would therefore mean selecting on a benchmark whose structural shape (one transition, no recurrent state to exploit) is not the operational shape. Second, deployability of the alternatives: The Static MLP cannot be deployed at T = 120 by construction. Mean-pooling 120 frames discards all temporal information, and concatenation produces a 5040-dimensional input that exceeds the TEE memory budget. So, even on the strictest reading of Table 1, the Static MLP’s T = 2 lead does not translate into a viable deployment option. Third, quantization maturity: The TensorFlow Lite Micro INT8 toolkit, targeted by the OP-TEE Trusted Application of Section 2, has more mature INT8 kernel support for LSTM than for GRU. The additional gating reset operation in GRU introduces a measurable post-quantization error that complicates deployment on the constrained-precision target. Fourth, framework independence: The Edge AI-Oracle architecture is independent of the specific recurrent cell choice. The TEE attestation, IPFS evidence log, smart contract pipeline, and Watchtower audit protocol remain valid under any temporal-classification head, so the LSTM-versus-GRU question is an engineering choice within the framework rather than a load-bearing claim of the contribution. Taken together, the LSTM choice is not a claim of uniform superiority across all evaluation conditions. It is the cell that best matches the deployment regime, the deployable alternative on that regime, the more quantization-friendly option for the constrained-precision target, and a substitutable component within an architecture whose contribution lies elsewhere.

The cross-dataset evaluation on BioVid Part A clarifies the architectural reach of the proposed pipeline beyond the synthetic SynPAIN corpus. Three observations from the BioVid LOSO results, none of which contradict the architectural claims of the present work, deserve explicit attention in the context of tele-rehabilitation deployment. First, the F1-score falloff from 0.722 (SynPAIN image-pair) to 0.519 (BioVid LOSO) reflects the genuine difficulty of recognizing subtle micro-reactions to calibrated thermal stimuli in healthy volunteers, in contrast to the more pronounced pain prototypes synthesized by the SynPAIN generation pipeline. The gap is consistent with the broader observation in the affective computing literature that recognition performance on synthetic facial corpora systematically overestimates performance on naturally elicited expressions. Second, the per-fold F1 distribution on BioVid is markedly bimodal: a subset of 18 of 174 LSTM evaluations (10.3%) yield F1 = 0.000, indicating subjects whose facial response to nociceptive stimulation is reduced or absent. This non-expressive subjects phenomenon is well documented for BioVid and represents a fundamental characteristic of natural pain expression rather than a methodological deficiency. The implication for tele-rehabilitation deployment is that any expression-based assessment system must be complemented by clinical workflow safeguards (periodic in-person verification, multi-modal physiological cues) for subjects whose facial signal is insufficiently expressive. Third, the cross-seed standard deviation of 0.017 to 0.024 is more than an order of magnitude smaller than the per-fold standard deviation of approximately 0.25 across subjects. Subject identity dominates the variability of recognition performance, which provides empirical justification for the strict leave-one-subject-out protocol adopted in the present work and argues against k-fold protocols that mix subjects across training and evaluation partitions.

A broader implication concerns the choice of facial expression as the assessment modality. Pain is a multidimensional experience with sensory, cognitive, emotional, and physiological components, and facial expression captures only its observable behavioural channel. The non-expressive subjects observed on BioVid are a concrete manifestation of this limitation, since a subset of individuals exhibit reduced or absent facial responses to nociceptive stimulation while still experiencing pain. The proposed architecture should therefore be understood as providing a single, privacy-preserving, and verifiable modality rather than a complete measurement of the pain experience. For non-expressive individuals and for clinical contexts where facial signal alone is insufficient, the assessment must be complemented by safeguards at the clinical-workflow level, such as periodic in-person verification, and ideally by additional modalities. The multi-modal extensions discussed below, which fuse the facial stream with skeletal motion or with the physiological signals available in BioVid, are the natural route to capturing the components of pain that facial expression does not convey, at the cost of a redesigned attestation boundary.

The nested hyperparameter search reported in Section 3 reinforces this interpretation. Because tuning under a leakage-free protocol did not recover the performance gap, the lower BioVid F1 is best understood as a property of the synthetic-to-real domain shift rather than a tractable configuration problem, which in turn motivates the multi-modal and dual-stream extensions discussed below as the more promising route to higher real-world accuracy.

The feature-level sanity check provides empirical support for the biomechanical validity of the proposed feature space. The direction and relative magnitude of the inter-class distance differences are consistent with the published biomechanical literature on facial pain expression [3,6], in which compression of the upper face and tightening of the lip-nose region are the dominant motor responses to nociceptive stimuli. This concordance constitutes empirical evidence that the proposed pipeline learns features semantically aligned with the clinical PSPI vocabulary rather than spurious correlates of the synthetic generation process.

The on-device measurements quantify what TEE isolation costs in practice. Even with worst-case SMC overhead, the total per-frame budget retains a several-fold margin below the 33 ms real-time envelope (Table 10). This leaves headroom for hardware-backed signing paths, whose latency characteristics on mobile secure elements [24,26] are an order of magnitude slower than the software path measured here.

The smart contract economics validate the dual-layer storage strategy for blockchain-anchored medical pipelines. The 24 h challenge window is conservative and can be tuned per deployment, trading finalization speed against the security margin for late disputes.

The per-transaction gas analysis above captures only the on-chain component of cost. A realistic assessment of economic feasibility must also account for off-chain infrastructure, clinical operational overhead, and integration with existing hospital systems, summarized in Table 11. The dominant on-chain finding is robust to gas-price volatility: the dual-layer storage strategy yields a 23.4-fold reduction in submission gas independent of the prevailing gas price, while the absolute fiat cost of a mainnet submission ranges from approximately 0.53 USD at 1 gwei to approximately 16 USD at a busier 30 gwei (3300 USD per ETH). Because continuous monitoring during a multi-week course would be economically sensitive to this range, the deployment-realistic configuration anchors on a Layer-2 rollup, where the per-submission cost falls to approximately 0.005 USD and is largely insulated from mainnet gas spikes. Off-chain costs are modest by comparison and are reported here as illustrative, order-of-magnitude figures rather than vendor quotes. IPFS pinning of session evidence logs costs approximately 0.10 USD per patient-month (illustrative, order of magnitude), a figure that follows from the computed canonical evidence-log size of approximately 0.37 GB per patient-month, corresponding to twenty 30 min sessions recorded at 24 fps with the 42-dimensional feature sequence, at commodity pinning rates. A single institution-operated Watchtower auditor runs on a commodity single cloud instance of one to two vCPUs at approximately 10 to 20 USD per month (illustrative). Against these figures, the principal recurring expense of a real deployment is not the decentralized infrastructure but the clinical operational overhead common to all tele-rehabilitation systems, namely clinician review time and the one-time engineering cost of integrating the on-chain audit trail with hospital information systems and insurance back ends. Relative to existing centralized platforms such as Kaia Health, SWORD Health, and Hinge Health, which carry comparable clinical operational costs but provide no decentralized audit trail, the proposed architecture adds a bounded and predictable infrastructure cost in exchange for cryptographic verifiability and tamper-evidence.

The practical deployment of the architecture must also account for the operational complexity of combining four subsystems, namely on-device Edge AI inference, a TEE, blockchain anchoring, and IPFS storage. Each layer scales differently. The on-device inference and TEE attestation scale per patient device and add no central cost, the IPFS evidence storage scales with the number and length of sessions, and the on-chain anchoring scales with submission frequency but is bounded by the per-transaction cost analysed below. The Watchtower is the only component requiring continuously provisioned central infrastructure, and a single institution-operated auditor is sufficient for the threat model considered here. A realistic first clinical pilot can reduce this complexity further by anchoring exclusively on a Layer-2 rollup, by operating a single Watchtower, and by treating the on-chain layer as an audit trail rather than a real-time dependency, which decouples clinical operation from blockchain latency. The principal integration burden in practice is connecting the on-chain audit trail to existing hospital information systems and insurance back ends, which is an engineering rather than an architectural obstacle and is the natural subject of a deployment study.

The adversarial tampering experiment demonstrates that the asynchronous Watchtower audit reliably detects large-magnitude tampering but does not, by itself, close the Oracle problem. Under an adaptive adversary the protocol bounds rather than eliminates patient-side fraud: it catches any inflation exceeding the dispute threshold, while small-magnitude or threshold-aware manipulation below δ ≈ 0.15 remains undetected, because the threshold cannot be tightened below the legitimate quantization-drift band without raising the false-positive rate. The residual surface therefore comprises three categories addressed at adjacent layers. First, sub-threshold inflation by a rational fraudster, which is the principal motivation for migrating the audit to a zero-knowledge proof-of-inference scheme (Section 4, future work) that removes the dispute threshold entirely. Second, physical presentation attacks, in which the patient voluntarily induces a pain expression in front of the legitimate camera. These lie outside the scope of any expression-based assessment system and must be addressed by the broader clinical workflow or by liveness-detection primitives. Third, model-evasion attacks that simultaneously fool the device-side and Watchtower reference models, which require white-box access to both variants and are unavailable to patient-class adversaries.

The per-ethnicity analysis revealed a residual demographic gap of approximately 8.5 percentage points in F1 between the strongest group (Caucasian, 0.765) and the weakest group (Black, 0.680). This gap is below the 10-percentage-point heuristic commonly cited as indicative of substantial bias, but it is systematic rather than seed-dependent, because the per-seed standard deviations (at most 0.021 for all ethnicities) are an order of magnitude smaller than the inter-group gap. Two observations clarify the nature of the gap. First, the AUC values are markedly more uniform across ethnicities (0.731 to 0.820) than the threshold-dependent F1-scores, which indicates that the disparity arises substantially from a single shared decision threshold being suboptimal for some groups rather than from a fundamental loss of discriminative capacity in the underlying scores. Per-ethnicity threshold calibration is therefore expected to narrow the operating-point component of the gap. Second, because the gap is measured on synthetic SynPAIN identities, its magnitude on real patient populations is unknown, and the BioVid corpus carries no ethnicity metadata with which to validate it cross-dataset, which leaves cross-dataset fairness an open question. Independently of the operating-point question, the gap warrants explicit mitigation in any production deployment. Candidate strategies include per-group decision-threshold calibration, demographic-stratified data augmentation during training, and active acquisition of additional clinical recordings from underrepresented groups, with the choice among them depending on whether the deployed gap is dominated by the operating point or by the representation.

One methodological choice that needs justification is the evaluation corpus. SynPAIN is synthetic, generated through commercial generative AI pipelines and validated through AU analysis [35]. The synthetic origin might at first look like a weakness for clinical validity, but the present work is scoped as an architectural and protocol-level validation, not a clinical efficacy study of the recognition model itself. Architectural claims rest on the structural properties of the data flow, not on absolute accuracy against real clinical inputs. Three further reasons argue for SynPAIN: the legacy UNBC-McMaster Archive is no longer available for new academic acquisitions, SynPAIN balances demographics across five ethnicities far better than legacy clinical datasets do, and a synthetic origin removes the ethical and privacy concerns of redistributing facial biometric data from real patients. The cross-dataset evaluation on BioVid Part A complements this synthetic-only validation with empirical evidence on real human pain expressions captured under realistic recording conditions.

The study has three limitations worth stating up front. First, the TEE-isolated configuration was characterized through a CPU-only surrogate benchmark with SMC overhead taken from the published literature, not through deployment on physical OP-TEE hardware. The surrogate is configured to be conservative (GPU disabled and single-thread execution approximate the worst-case throughput of an OP-TEE secure partition), so it gives a lower-bound estimate of on-device performance rather than a best case, but a definitive production-deployment characterization needs a study on an actual ARM TrustZone platform. Second, the Watchtower protocol assumes a single trusted off-chain auditor run by the medical institution. A fully decentralized variant would need a multi-party validator committee, or a replacement of the asynchronous audit by a zero-knowledge proof of inference. Third, the per-ethnicity breakdown on SynPAIN shows a residual gap of 8.5 percentage points between the strongest and weakest groups, which calls for explicit mitigation in any production deployment as discussed above. The BioVid corpus does not contain ethnicity metadata and therefore could not extend this fairness analysis, leaving cross-dataset fairness validation as an open direction.

Three directions for further research follow from these limitations. The first is to migrate the asynchronous audit to a Zero-Knowledge Machine Learning (zkML) framework. Recent succinct cryptographic proof constructions for neural network inference [23] suggest that the LSTM inference of the present work could be accompanied by a constant-size proof of correct execution, verifiable on-chain at constant gas. This would eliminate the 24 h challenge window and replace it with mathematical certainty. The main obstacle today is the proof-generation time of zkML systems, which exceeds the per-frame budget of an edge device by several orders of magnitude. The second direction is to explore architectural alternatives that exploit the regime-dependence and dataset-dependence findings documented above: a hybrid deployment that combines a feed-forward Static MLP head for short-context spot-check assessments with a recurrent head for continuous monitoring may yield better aggregate performance than either head alone. A complementary architectural extension is the fusion of the facial stream with synchronized skeletal motion of the patient during the rehabilitation exercise. A recently proposed dual-stream framework [14] combines YOLO11-based person and face detection with pose estimation and facial expression recognition, coupled through a bidirectional LSTM that jointly models temporal dependencies in the skeletal and facial streams, and reports an F1-score of 0.89 on multi-modal state assessment in a rehabilitation-oriented setting. Embedding an analogous dual-stream design into the Edge AI-Oracle architecture proposed here would allow the pain estimate to be cross-referenced against the motor execution of the prescribed exercise, which is the clinically natural context of tele-rehabilitation. Multi-modal fusion with physiological signals available in BioVid (galvanic skin response, electrocardiogram, electromyography) constitutes a further architectural extension that may improve recognition performance, although such fusion, like the dual-stream extension above, would require redesign of the TEE attestation boundary and is therefore outside the scope of the current decentralized audit framework. The third direction is the deployment of the proposed Trusted Application on physical ARM TrustZone development hardware, complementing the surrogate-based latency characterization presented here, together with extension to patient populations under appropriate clinical protocols.

## 5. Conclusions

The urgent problem of objective and tamper-resistant pain syndrome assessment in decentralized tele-rehabilitation systems is addressed through the proposed Edge AI-Oracle architecture. By combining on-device spatiotemporal inference with hardware-rooted cryptographic attestation and asynchronous decentralized audit, the work shows that privacy, economic feasibility, and tamper-evidence can be achieved simultaneously within a single architecture, and it characterizes honestly the two dimensions that remain open, namely recognition accuracy on real-world data and robustness to small-magnitude tampering below the dispute threshold.

The scientific novelty of the obtained results consists in the following. A decentralized tele-rehabilitation architecture is proposed that, to our knowledge among existing tele-rehabilitation systems, runs the spatiotemporal pain recognition pipeline (MediaPipe Face Mesh combined with a recurrent classifier deployed as a two-layer LSTM network) inside a hardware-isolated TEE on the patient’s edge device. The architectural validity of the framework is established through a comparative empirical evaluation of feed-forward and recurrent classifiers across two temporal regimes on the synthetic SynPAIN corpus, and through cross-dataset validation on the BioVid Part A corpus of real human pain expressions, where the recurrent architectures attain statistically equivalent performance under the strict leave-one-subject-out evaluation protocol across 87 subjects. The inference latency budget was estimated through a CPU-only surrogate benchmark. The architecture guarantees confidentiality of biometric data through the Zero-Video Transmission principle, and verifiability of the inference result through ECDSA secp256k1 attestation, so that raw video never has to leave the device.

The Oracle problem for facial pain assessment is partially addressed by an asynchronous Watchtower protocol with a Timelock-based optimistic verification window. An adaptive-adversary analysis shows that the protocol reliably detects gross score tampering but bounds rather than eliminates fraud, since inflation below the dispute threshold (δ ≈ 0.15) evades detection. Closing this residual gap is the principal motivation for the zero-knowledge proof-of-inference direction outlined below.

The practical significance of the obtained results lies in the experimental confirmation that the architecture fits within the resource budget of a current mobile rehabilitation client. The hybrid spatiotemporal pipeline reaches an operating-point F1-score of 0.72 on the SynPAIN evaluation corpus but only 0.52 on the BioVid Part A leave-one-subject-out evaluation, a gap that reflects the synthetic-to-real distribution shift and that marks recognition accuracy on genuine pain expressions as the principal open problem for clinical use. Within this scope the on-device budget is comfortable, with a 0.34 MB model footprint and a per-inference latency of about 0.45 ms in the INT8-quantized configuration on the deployment-relevant T = 120 sliding window, as estimated by a CPU-only surrogate in place of physical ARM TrustZone hardware. The smart contract layer consumes 160,261 gas per submission, a 23.4-fold reduction relative to naive on-chain recording that holds independent of gas price. In fiat terms this is an illustrative 0.53 USD on Ethereum mainnet at 1 gwei, rising to roughly 16 USD at a busier 30 gwei (3300 USD per ETH), and falling to approximately 0.005 USD on a Layer-2 rollup such as Arbitrum One, which is the deployment-realistic configuration for continuous monitoring. This provides a basis for connecting automated medical assessment to parametric insurance smart contracts and decentralized clinical audit trails, which are operational requirements of Health 4.0 deployments.

Prospects for further research are three-fold. The first is migration of the asynchronous audit to a zkML framework based on succinct proofs of LSTM inference, which would replace the 24 h challenge window with instant on-chain mathematical verification and, critically, would close the residual sub-threshold tampering gap identified in the adaptive-adversary analysis by removing the dispute threshold entirely. The second is deployment of the proposed Trusted Application on physical ARM TrustZone development hardware, to complement the surrogate-based latency characterization presented here. The third is the exploration of architectural extensions, including hybrid recognition heads that combine feed-forward and recurrent components (motivated by the regime-dependence and dataset-dependence findings documented in the present study), and of recognition performance.

## Figures and Tables

**Table 1:** Pain recognition performance on the SynPAIN test split (image-pair pipeline, five-fold identity-disjoint cross-validation, mean ± std). The convolutional baselines (ResNet-50 and MobileNetV3-Small) are scored at the crop level, consistent with their training regime, whereas the landmark-based and recurrent models are scored at the composite level. F1 is the primary comparison metric across rows because it is robust to this aggregation difference.

Model Architecture	n_params	Accuracy	F1-Score	Precision	Recall	AUC
Geometric centroid (no learning)	0	0.684 ± 0.017	0.695 ± 0.009	0.683 ± 0.025	0.707 ± 0.010	0.774 ± 0.019
ResNet-50 frozen + class weights	24 M	0.835 ± 0.014	0.729 ± 0.041	0.629 ± 0.036	0.867 ± 0.058	0.925 ± 0.012
MobileNetV3-Small frozen + class weights	1.0 M	0.778 ± 0.015	0.647 ± 0.018	0.545 ± 0.025	0.796 ± 0.040	0.860 ± 0.020
Static MLP, expressive only (V1)	9 K	0.758 ± 0.034	0.765 ± 0.049	0.751 ± 0.011	0.784 ± 0.098	0.845 ± 0.039
Static MLP, neutral + expressive (V2)	12 K	0.777 ± 0.022	0.794 ± 0.022	0.748 ± 0.027	0.848 ± 0.041	0.860 ± 0.027
Hybrid GRU (10 seeds)	47,809	0.763 ± 0.020	0.778 ± 0.020	0.744 ± 0.026	0.816 ± 0.040	0.845 ± 0.022
Hybrid LSTM (deployment, 10 seeds) *	62,529	0.715 ± 0.025	0.722 ± 0.036	0.717 ± 0.038	0.735 ± 0.085	0.790 ± 0.025

* Effect size for the Hybrid LSTM versus Hybrid GRU comparison on this regime: Cohen $d_{z}$ = −1.59 (large), 95 percent Confidence Interval (CI) on ΔF1 [−0.066, −0.046], favouring GRU.

**Table 2:** Per-ethnicity recognition performance of the deployment Hybrid LSTM (10 seeds × 5 folds, mean ± std at decision threshold 0.5).

Ethnicity	n	Accuracy	F1-score	Precision	Recall	AUC
Black	1009	0.673 ± 0.008	0.680 ± 0.015	0.699 ± 0.011	0.663 ± 0.032	0.731 ± 0.006
Caucasian	1032	0.723 ± 0.014	0.765 ± 0.008	0.681 ± 0.019	0.873 ± 0.024	0.796 ± 0.012
East Asian	1047	0.749 ± 0.013	0.739 ± 0.021	0.754 ± 0.017	0.726 ± 0.047	0.820 ± 0.010
Middle Eastern	1178	0.723 ± 0.010	0.735 ± 0.013	0.727 ± 0.016	0.744 ± 0.030	0.793 ± 0.009
South Asian	1040	0.704 ± 0.011	0.688 ± 0.019	0.713 ± 0.008	0.666 ± 0.035	0.780 ± 0.007

**Table 3:** Recurrent cell comparison on the BioVid Part A binary task (BL1 vs. PA4) under leave-one-subject-out cross-validation. Aggregated across 2 seeds and 87 folds, totalling 174 paired observations per architecture.

Architecture	Accuracy	F1-Score	Precision	Recall	AUC
Hybrid LSTM *	0.579 ± 0.135	0.519 ± 0.256	0.561 ± 0.258	0.578 ± 0.344	0.640 ± 0.198
Hybrid GRU	0.587 ± 0.138	0.499 ± 0.267	0.584 ± 0.265	0.531 ± 0.342	0.637 ± 0.195
Δ (GRU − LSTM)	+0.008	−0.020	+0.023	−0.047	−0.003
Statistical test	Wilcoxon signed-rank				
W statistic	7214				
*p*-value	0.549				
Status at α = 0.05	not significant				

* Effect size for the Hybrid LSTM versus Hybrid GRU comparison: matched-pairs rank-biserial r = 0.06 (negligible), 95 percent CI on ΔF1 [−0.008, 0.049].

**Table 4:** Recurrent cell comparison across temporal regimes: image-pair (T = 2, n = 50 paired observations from 10 seeds × 5 folds) versus video leave-one-stratum-out (LOSO) (T = 120, n = 100 paired observations from 5 seeds × 20 strata).

Architecture	T = 2 F1	T = 2 AUC	T = 120 Mean F1	T = 120 Median F1	T = 120 AUC
Hybrid LSTM	0.722 ± 0.036	0.790 ± 0.025	0.683 ± 0.400	1.000	0.880 ± 0.327
Hybrid GRU	0.778 ± 0.020	0.845 ± 0.022	0.650 ± 0.368	0.667	0.840 ± 0.369
Δ (GRU − LSTM)	+0.056	+0.055	−0.033	−0.333	−0.040
Statistical test	paired *t*-test		Wilcoxon signed-rank		
*p*-value	3.3 × 10^−15^		0.167		
Status at α = 0.05	significant		not significant		

**Table 5:** Temporal-window sensitivity on the SynPAIN video regime (leave-one-stratum-out, 5 seeds, per-stratum aggregation, n = 100 paired observations). All other hyperparameters held fixed.

Window T (Frames)	Context at 24 fps	Mean F1	Mean AUC
30	1.25 s	0.707	0.900
60	2.50 s	0.713	0.880
90 *	3.75 s	0.867	0.910
120 (deployed)	5.00 s	0.723	0.860

* The T = 90 window attains the highest F1 and AUC on this corpus, exceeding the deployed T = 120 window by ΔF1 = 0.143 (Wilcoxon signed-rank, *p* = 7.0 × 10^−4^). T = 120 is retained as the deployed window for the reasons given in the text. The T = 120 value here (0.723) differs slightly from the 0.683 reported in Table 4 due to run-to-run non-determinism on the 40-clip corpus and does not affect the relative comparison across window lengths, which uses identical seeds throughout.

**Table 6:** On-device performance budget under Trusted Execution Environment (TEE) surrogate conditions at the T = 120 deployment window (Apple Silicon ARM64, 1000 trials, single-thread Central Processing Unit (CPU), Graphics Processing Unit (GPU) disabled).

Performance Metric	FP32	INT8 (TFLite)
LSTM inference latency, median (p50)	1.96 ms	0.45 ms
LSTM inference latency, p95	2.69 ms	0.47 ms
LSTM inference latency, p99	3.11 ms	0.48 ms
Model parameters	60,481	60,481
Model file size	0.27 MB (h5)	0.34 MB (tflite)
ECDSA secp256k1 signing latency, mean	707 µs	-
ECDSA secp256k1 signing latency, p99	2052 µs	-
Estimated SMC overhead per call (literature [24,25])	0.5–2.0 ms	0.5–2.0 ms
Total budget per inference (worst-case sum)	~4.7 ms	~3.2 ms

**Table 7:** Decentralized infrastructure performance: smart contract gas profile and Watchtower audit calibration.

Component	Metric	Value
Smart contract (Solidity 0.8.24, optimizer 200 runs)	submitPainData (proposed, IPFS-anchored)	160,261 gas
	submitPainData (baseline, full on-chain payload)	3,744,872 gas
	raiseDispute	30,927 gas
	executeData	30,531 gas
	Gas reduction factor (proposed vs. baseline)	×23.4
	Illustrative cost of submitPainData on L1 (1 gwei, 3300 USD/ETH, April 2026)	0.53 USD
	Illustrative cost of submitPainData on L1, 1 to 30 gwei	0.53 to 16 USD
	Illustrative cost of baseline submitPainData on L1	12.36 USD
	Illustrative cost of submitPainData on Arbitrum One (post-EIP-4844)	~0.005 USD
Watchtower discrepancy distribution	Legitimate (n = 500), mean |Δ|	0.027
	Legitimate, p95 |Δ|/max |Δ|	0.065/0.131
Watchtower detection, adaptive adversary, TPR at thresholds 0.1, 0.2, 0.3, 0.5 *	inflation 0.10	0.00, 0.00, 0.00, 0.00
	inflation 0.20	1.00, 0.00, 0.00, 0.00
	inflation 0.30	1.00, 1.00, 1.00, 0.00
	inflation 0.50	1.00, 1.00, 1.00, 0.00
	FPR on legitimate set	0.006, 0.000, 0.000, 0.000

* In the Watchtower detection rows, each cell lists the true-positive rate at dispute thresholds of 0.1, 0.2, 0.3 and 0.5 respectively, and the final row reports the corresponding false-positive rate on the legitimate set.

**Table 8:** Inference latency comparison of evaluated models, measured under TEE surrogate conditions on Apple Silicon ARM64, single-process Python, n_warmup = 50, n_trials = 1000. Latencies are reported as the median (p50) over 1000 trials.

Model	Architecture Type	p50 Latency	p95 Latency	Throughput (FPS)	Suitability for Edge Deployment
Geometric centroid	Landmark, no learning	0.002 ms	0.002 ms	648,929	Lower-bound reference
Static MLP V2	Landmark, frame-level	0.072 ms	0.083 ms	14,643	Spot-check only
Hybrid LSTM INT8 (T = 120)	Landmark, recurrent	0.45 ms	0.47 ms	2240	Proposed deployment
Hybrid LSTM FP32 (T = 120)	Landmark, recurrent	1.96 ms	2.69 ms	511	Suitable
Hybrid GRU FP32 (T = 120)	Landmark, recurrent	2.17 ms	2.406 ms	460	Suitable
MobileNetV3-Small + head	CNN, lightweight	3.42 ms	3.873 ms	292	Outside TEE memory budget
ResNet-50 + head	CNN, heavy	27.16 ms	29.912 ms	37	Cloud-class only

**Table 9:** Summary of architectural trade-offs across model categories. F1-scores aggregated from Table 1, Table 3 and Table 4. Computational cost is interpreted as parameter count combined with inference latency from Table 8.

Model Type	Temporal Modelling	Input Representation	Computational Cost	Robustness (F1 Across Regimes)	Edge Suitability
Static MLP V2	None	Landmarks	Very low (12 K params)	Highest on T = 2 (0.794), N/A on T = 120	High (spot-check)
Hybrid GRU	Single-gate recurrent	Landmarks	Low (48 K params)	Strong on T = 2 (0.778), dataset-dependent on T = 120	High
Hybrid LSTM (proposed)	Dual-state recurrent	Landmarks	Low (62,529 params)	Dataset-dependent on T = 2 (0.722) and T = 120 (0.683 SynPAIN, 0.519 BioVid)	High
MobileNetV3-Small	None	Images	Medium (1 M params)	Moderate (0.647)	Outside TEE budget
ResNet-50	None	Images	High (24 M params)	Competitive (0.729) but memory-heavy	Cloud-class only

**Table 10:** Consolidated per-frame latency budget of the deployment Hybrid LSTM INT8 pipeline at T = 120 against the 33 ms real-time interaction envelope (30 fps).

Pipeline Stage	Latency	Source
MediaPipe Face Mesh landmark extraction (Normal World)	4.5 ms	measured, Section 2
LSTM INT8 inference (Secure World)	0.45 ms	measured, Table 6
ECDSA secp256k1 signing (Secure World)	0.71 ms	measured, Table 6
SMC cross-world overhead (worst case)	≤2.0 ms	literature [24,25]
Total per-frame latency	≤7.7 ms	sum
Real-time interaction envelope (30 fps)	33 ms	target
Headroom factor	≥4.3×	derived

**Table 11:** Total cost-of-ownership components of the proposed architecture, separated by scaling behaviour. On-chain figures use April 2026 reference parameters (3300 USD per ETH).

Cost Component	Scaling Unit	Estimated Cost	Notes
On-chain submission, L1	per submission	0.53 to 16 USD	1 to 30 gwei range, 23.4× below naive on-chain baseline
On-chain submission, L2 (Arbitrum)	per submission	~0.005 USD	deployment-realistic, gas-spike insulated
IPFS evidence pinning	per patient-month	~0.10 USD (illustrative)	≈0.37 GB/patient-month computed log size, commodity pinning rates, order-of-magnitude
Watchtower auditor hosting	per month	~10 to 20 USD (illustrative)	single commodity 1 to 2 vCPU cloud instance, institution-operated
On-device inference and TEE	per device	none (patient hardware)	no central cost, scales with patient count
Hospital-system integration	one-time	engineering, deployment-specific	connection to HIS and insurance back ends
Clinician review	per session	common to all tele-rehab	not specific to this architecture

## Data Availability

The source code and trained model weights are not publicly available, as they constitute proprietary intellectual property of the authors and form part of a system that is still under active development and testing. The experimental protocols, model architectures, hyperparameters, random seeds, and evaluation procedures are described in detail in Section 2 to support independent re-implementation of the methodology. The SynPAIN dataset is openly available via its original release [35], and the BioVid Heat Pain Database, Part A, is available from the original data providers under their access procedure [36,37]. No new datasets were generated in this study.

## Abbreviations

The following abbreviations are used in this manuscript:

AU	Action Unit
AUC	Area Under the Curve
CID	Content Identifier
CNN	Convolutional Neural Network
CV	Computer Vision
ECDH	Elliptic Curve Diffie–Hellman
ECDSA	Elliptic Curve Digital Signature Algorithm
EVM	Ethereum Virtual Machine
FACS	Facial Action Coding System
FPR	False-Positive Rate
FPS	Frames Per Second
GDPR	General Data Protection Regulation
GRU	Gated Recurrent Unit
HIPAA	Health Insurance Portability and Accountability Act
IMU	Inertial Measurement Unit
IPFS	InterPlanetary File System
JSON	JavaScript Object Notation
LOSO	Leave-One-Subject-Out (also Leave-One-Stratum-Out)
LSTM	Long Short-Term Memory
MLP	Multi-Layer Perceptron
MSK	Musculoskeletal
NPRS	Numeric Pain Rating Scale
OP-TEE	Open Portable Trusted Execution Environment
OS	Operating System
PSPI	Prkachin and Solomon Pain Intensity
RAM	Random Access Memory
ReLU	Rectified Linear Unit
ROC	Receiver Operating Characteristic
SHA	Secure Hash Algorithm
SMC	Secure Monitor Call
TA	Trusted Application
TEE	Trusted Execution Environment
TFLite	TensorFlow Lite
TPR	True-Positive Rate
VAS	Visual Analog Scale
zkML	Zero-Knowledge Machine Learning

## Appendix A

This appendix details the 42 geometric features summarized in Section 2. Table A1 lists, for each of the six PSPI Action Units, the seven inter-landmark Euclidean distances of Equation (5), the MediaPipe 478-point FaceLandmarker indices of each landmark pair, and the anatomical structure that the distance samples. AU7 (lid tightener) and AU43 (eye closure) share several eyelid-aperture pairs, because both Action Units are governed by the same palpebral landmarks.

**Table A1:** The 42 geometric features of Equation (5): six PSPI Action Units, each defined by seven Euclidean distances between MediaPipe 478-point landmark pairs, with the anatomical structure each distance samples.

AU	Landmark Pair (MediaPipe 478-Point)	Anatomical Interpretation
AU4 (Brow Lowerer)	(107, 159)	Inner brow to upper eyelid, brow-eyelid vertical gap
AU4	(336, 386)	Inner brow to upper eyelid (contralateral)
AU4	(107, 336)	Inner brow to inner brow, inter-brow glabellar distance
AU4	(105, 133)	Eyebrow to medial eye corner, brow-to-canthus height
AU4	(334, 362)	Eyebrow to medial eye corner (contralateral)
AU4	(9, 1)	Glabella to nose base, central brow-to-nose vertical span
AU4	(70, 300)	Outer eyebrow to outer eyebrow, lateral brow span
AU6 (Cheek Raiser)	(205, 145)	Cheek to lower eyelid, cheek elevation toward eye
AU6	(425, 374)	Cheek to lower eyelid (contralateral)
AU6	(205, 61)	Cheek to mouth corner, cheek-to-mouth distance
AU6	(425, 291)	Cheek to mouth corner (contralateral)
AU6	(145, 61)	Lower eyelid to mouth corner, mid-face vertical compression
AU6	(374, 291)	Lower eyelid to mouth corner (contralateral)
AU6	(205, 425)	Cheek to cheek, inter-cheek width
AU7 (Lid Tightener)	(159, 145)	Upper to lower eyelid, palpebral aperture
AU7	(386, 374)	Upper to lower eyelid (contralateral)
AU7	(33, 133)	Lateral to medial eye corner, palpebral fissure width
AU7	(263, 362)	Lateral to medial eye corner (contralateral)
AU7	(158, 153)	Upper to lower eyelid (secondary points)
AU7	(385, 380)	Upper to lower eyelid (secondary, contralateral)
AU7	(468, 473)	Iris centre to iris centre, inter-iris distance (478-point iris reference)
AU9 (Nose Wrinkler)	(1, 9)	Nose base to glabella, naso-frontal vertical span
AU9	(168, 1)	Nasal bridge to nose base, nasal dorsum length
AU9	(49, 279)	Nostril wing to nostril wing, alar width
AU9	(1, 0)	Nose base to upper lip, sub-nasal philtrum distance
AU9	(9, 0)	Glabella to upper lip, central mid-face span
AU9	(49, 61)	Nostril wing to mouth corner, nasolabial distance
AU9	(279, 291)	Nostril wing to mouth corner (contralateral)
AU10 (Upper Lip Raiser)	(0, 1)	Upper lip to nose base, upper-lip elevation toward nose
AU10	(0, 17)	Upper lip to lower lip (outer), vertical mouth gap
AU10	(61, 291)	Mouth corner to mouth corner, mouth width
AU10	(0, 152)	Upper lip to chin, lower-face vertical span
AU10	(61, 49)	Mouth corner to nostril wing, nasolabial fold
AU10	(291, 279)	Mouth corner to nostril wing (contralateral)
AU10	(0, 14)	Outer upper lip to inner lower lip, lip aperture
AU43 (Eyes Closed)	(159, 145)	Upper to lower eyelid, eyelid-closure aperture
AU43	(386, 374)	Upper to lower eyelid (contralateral)
AU43	(158, 153)	Upper to lower eyelid (secondary points)
AU43	(160, 144)	Upper to lower eyelid (secondary points)
AU43	(385, 380)	Upper to lower eyelid (secondary, contralateral)
AU43	(387, 373)	Upper to lower eyelid (secondary, contralateral)
AU43	(159, 468)	Upper eyelid to iris centre, lid-to-iris closure (478-point iris reference)
